# Fluence-Dependent Effects of 940-nm Photobiomodulation on the Proliferation and Osteoblastic Differentiation of Human Periodontal Ligament Stem Cells

**DOI:** 10.3390/dj14100676

**Published:** 2026-10-16

**Authors:** Jovan Marković, Milan Marković, Jelena Đokić, Sergej Tomić, Luka Pavlović, Mile Eraković, Zorana Stamenković, Miodrag Čolić

**Affiliations:** 1 Clinic for Orthodontics, School of Dental Medicine, University of Belgrade, 11000 Belgrade, Serbia; jovan.markovic@stomf.bg.ac.rs (J.M.); zorana.stamenkovic@stomf.bg.ac.rs (Z.S.); 2 Institute for the Application of Nuclear Energy, University of Belgrade, 11000 Belgrade, Serbia; milan.markovic@inep.co.rs (M.M.); sergej.tomic@inep.co.rs (S.T.); luka.pavlovic@inep.co.rs (L.P.); 3 Institute of Molecular Genetics and Genetic Engineering, University of Belgrade, 11000 Belgrade, Serbia; jelena.djokic@imgge.bg.ac.rs; 4 Clinic for Stomatology, Medical Faculty of the Military Medical Academy, University of Defense, 11154 Belgrade, Serbia; erakovic.nino78@gmail.com; 5 Serbian Academy of Sciences and Arts, Knez Mihailova 35, 11000 Belgrade, Serbia; 6 Faculty of Medicine Foča, University of East Sarajevo, 73300 Foča, Bosnia and Herzegovina

**Keywords:** photobiomodulation, periodontal ligament stem cells, osteoblastogenesis, proliferation, gene expression

## Abstract

**Background/Objectives**: Photobiomodulation (PBM) has been proposed to regulate periodontal tissue remodeling, but its effects on osteoblastogenesis in periodontal ligament stem cells (PDLSCs) remain highly dependent on laser parameters. This study investigated the fluence-dependent effects of 940 nm diode laser irradiation on the proliferation and osteoblastic differentiation of human PDLSCs. **Methods**: Six primary human PDLSC cultures were established and characterized using flow cytometry and multilineage differentiation assays. Cell proliferation was assessed after a single irradiation at fluences of 1, 2, 4, and 10 J/cm^2^. Osteoblastogenesis was induced using repeated irradiation regimens initiated at 2, 4, or 10 J/cm^2^ under suboptimal or complete osteogenic conditions and evaluated by Alizarin Red staining on day 21. The expression of osteogenic genes (*RUNX2*, *BMP2*, *SPP1*, *TGFB1*, *COL1A1*, *ALPL*, and *BGLAP*) was evaluated by qRT-PCR at different time points. Culture supernatants were analyzed for soluble osteogenic mediators. **Results**: The established PDLSC cultures had an MSC-like phenotype, robust osteogenic potential, moderate chondrogenic potential, and weak or no adipogenic potential. Proliferation of PDLSCs was considerably increased by PBM at 2 J/cm^2^ on day 7, whereas fluences of 4 and 10 J/cm^2^ inhibited proliferation. Osteoblastic mineralization was increased with the 2 J/cm^2^ irradiation regimen but was markedly reduced with the 10 J/cm^2^ regimen. Gene expression analysis confirmed this fluence-dependent response under optimal culture conditions. The 2 J/cm^2^ irradiation regimen significantly upregulated each analyzed osteogenic gene at one or more time points, whereas the 10 J/cm^2^ regimen downregulated *RUNX2*, *BMP2*, *SPP1*, *COL1A1*, and *BGLAP*. PBM at 2 J/cm^2^ also increased production of soluble BMP-2, OPG, PDGF-BB, ALP, and Leptin in a time-dependent manner. **Conclusions**: PBM exerts a fluence-dependent effect on human PDLSCs. A single irradiation at 2 J/cm^2^ increased proliferation, whereas the irradiation regimen initiated at 2 J/cm^2^ promoted osteoblastogenesis. In contrast, higher-fluence conditions, particularly the regimen initiated at 10 J/cm^2^, were inhibitory.

## 1. Introduction

Photobiomodulation (PBM) is a therapeutic approach that uses visible red or near-infrared light generated by a laser or a light-emitting diode (LED) to manage a wide range of medical conditions, including those in dentistry. PBM was formerly known as low-level laser therapy (LLLT), and both terms are now used interchangeably in the literature to emphasize that the light effect is biomodulatory rather than a destructive thermal effect on tissues. PBM effects are mediated by photosensitive cellular molecules, primarily cytochrome c oxidase in mitochondria and light-sensitive ion channels in the cell membrane. At the target level, laser energy is converted into metabolic energy via the respiratory chain, producing adenosine triphosphate (ATP), nitric oxide (NO), and reactive oxygen species (ROS). These primary messengers, together with increased Ca^2+^ flux as a secondary messenger, drive key biological processes: activation and regulation of transcriptional programs, cell proliferation, mitochondrial metabolism, and cell-specific functions [1,2,3]. The application of PBM in dentistry spans a broad spectrum of disorders and symptoms, including pain reduction and analgesia, management of tooth sensitivity, as well as improvement of bone and soft-tissue healing. PBM supports osseointegration, modulates peri-implant and periodontal inflammation, and manages temporomandibular joint (TMJ) disorders. The therapy also shows efficacy in regenerative endodontics and is used adjacently in mucogingival therapy, alveolar osteitis/dry socket management, and many other dental applications [4,5]. In periodontics, PBM has been investigated as an adjunctive approach to modulate inflammation, reduce postoperative discomfort, and support periodontal wound healing and regeneration. Its biological effects appear to depend strongly on irradiation parameters and the local tissue environment [4,5].

In orthodontics, PBM has gained increasing attention as an adjunctive therapy approach to accelerate orthodontic tooth movement (OTM) while also alleviating inflammation, reducing pain, and increasing tissue remodeling. OTM is a carefully regulated biological process involving bone resorption on the compression side and bone formation on the tension side of the periodontal ligament. Most evidence indicates that PBM may impact this remodeling process through interactions with mechanotransduction pathways. Photonic and mechanical signals converge on multiple cellular targets and are integrated across several signaling pathways, where they may reinforce or amplify one another [6,7].

This PBM–mechanotransduction crosstalk amplifies or modulates numerous cellular processes and intercellular communication. The most relevant mechanisms involve stimulation of osteoblasts and odontoblasts on the tension side and activation of osteoclasts on the compression side, primarily through interactions between stromal and immune cells within the periodontal ligament. These mechanisms include cell proliferation, differentiation, apoptosis, autophagy, extracellular matrix remodeling, angiogenesis, communication between stromal and immune cells, and cell-type-specific functional responses [6,7].

The periodontal ligament is a specialized connective tissue located between the tooth root and the adjacent bone. It comprises various cell populations, fibers, extracellular matrix, blood vasculature, and other components. Its primary role is to anchor the tooth to the alveolar bone, enable the tooth to withstand chewing forces, and absorb excess pressure from clenching and grinding. The periodontal ligament also facilitates tooth movement, supports tooth eruption, and promotes repair and regeneration of injured periodontal structures [8].

Periodontal ligament stem cells (PDLSCs) are a special type of dental mesenchymal stem cells (MSCs). They have the ability to self-renew and can also differentiate into osteoblasts, cementoblasts, and other mesenchymal-derived cells under appropriate culture conditions [9,10]. Mechanoreceptors are also expressed by PDLSCs to integrate mechanical and biochemical signals, a feature indispensable for OTM [7,10]. In particular, tensile stimulation promotes osteogenic differentiation of PDLSCs [7,11]. In contrast, excessive or abnormal mechanical stress may suppress their differentiation profile [7]. These features, in combination with the ease of culturing PDLSCs ex vivo, make them a useful in vitro model for studying osteoinduction [10,11]. This model is well suited to the controlled study of the effects of PBM alone or in combination with tensile strength on osteoblastic differentiation [12,13]. PDLSCs are also promising candidates for periodontal regeneration in the context of intensive osteodestructive processes associated with periodontitis and periapical lesions. At the same time, their ability to modulate inflammation, tissue remodeling, and regeneration makes them a promising therapeutic target in these diseases [11].

Numerous studies suggest that PBM can enhance the stemness and differentiation capacity of PDLSCs. However, there are significant differences in the results, which mostly depend on variations in key laser parameters. This is best illustrated by a recent systematic review by Mylona et al. [14], who analyzed 11 in vitro studies. The authors concluded that PBM generally promotes PDLSC proliferation, their differentiation to osteoblasts, and stimulation of mineralization. At the same time, they highlighted that the differences in results may be due to substantial heterogeneity among the irradiation protocols used, particularly in wavelength, fluence, and irradiation regimen [14]. Taken together, the available data indicate that an optimal PBM protocol for inducing osteoblastic differentiation of PDLSCs has yet to be clearly established [15]. It is important to emphasize that the review by Mylona et al. [14] provided evidence that 940 nm laser irradiation is less effective in PDLSC stimulation than light irradiation in the 630–830 nm range. This observation is particularly relevant because it indicates that wavelength selection may be a critical determinant of PBM efficacy in PDLSCs.

This recent review prompted us to extend our preliminary observations on 940 nm diode laser irradiation, which showed that PBM strongly promoted osteoblastic differentiation in our human PDLSC cultures under clearly defined fluence conditions. These observations challenged the view that this wavelength may be unsuitable for PBM protocols in osteoregenerative dentistry [14]. However, because the magnitude and direction of the PBM effect appeared to depend critically on dose parameters [14,15], we tested the hypothesis that laser fluence is a key determinant of PDLSC osteogenic commitment. Accordingly, the overall aim of this study was to evaluate the effects of 940 nm laser irradiation on PDLSC proliferation and osteogenic differentiation. The specific objectives were to assess: (i) the effect of different single-exposure fluences on PDLSC proliferation; (ii) the effect of different irradiation regimens on extracellular matrix mineralization; (iii) changes in the expression of early- and late-stage osteogenic genes; and (iv) changes in the production of soluble osteogenic factors associated with osteoblastic differentiation.

## 2. Materials and Methods

### 2.1. Study Design

This in vitro study on human PDLSCs was approved by the Ethical Committee of the Dental School, University of Belgrade (No. 36/35, dated 11 September 2023), and written consent was obtained from donors of extracted teeth. Teeth were extracted at the Dental School of the University of Belgrade for orthodontic reasons. There were 6 healthy donors (4 males and 2 females, aged 18–26 years). The extracted teeth included 5 maxillary premolars and one maxillary third molar. The donors were non-smokers and had no periodontitis, diabetes, systemic autoimmune diseases, or malignancies. They also did not receive antibiotics or immunosuppressive drugs for 2 months prior to tissue sampling. Other data from the medical histories of study participants were not recorded. All in vitro experiments were conducted at the Institute for the Application of Nuclear Energy (INEP) of the University of Belgrade, except for gene expression studies, which were conducted at the Institute for Molecular Genetics and Genetic Engineering of the University of Belgrade.

### 2.2. Establishment of Primary Donor-Derived PDLSC Cultures

The methodology for isolating and establishing PDLSCs was adapted from previously published protocols [16,17]. Immediately after extraction, teeth were transported to the laboratory in sterile phosphate-buffered saline containing an antibiotic–antimycotic solution. Teeth were processed under aseptic conditions in a biosafety cabinet. Root surfaces were washed several times with sterile phosphate buffer (PBS) to remove blood and debris before collecting periodontal ligament tissue. Tissue was gently scraped from the middle third of the root surface, avoiding the apical and cervical regions to minimize contamination by dental pulp, gingival tissue, or epithelial remnants. Collected tissue was minced into small fragments and subjected to enzymatic digestion in serum-free α-MEM medium containing collagenase type I (5 µg/mL) and dispase (40 IU/mL) (all components from Sigma-Aldrich, Darmstadt, Germany) for approximately two hours at 37 °C.

Following digestion, the enzyme activity was neutralized by adding complete culture medium containing α-MEM supplemented with 10% fetal calf serum (FCS) and antibiotics (1% penicillin and 1% gentamicin, both from Galenika a.d., Belgrade, Serbia). After incubation, the digested tissue fragments were pressed using a sterile syringe plunger. The resulting cell suspension, which also contained small undigested fragments, was subsequently washed by centrifugation in α-MEM medium. The isolated cells and tissue fragments were seeded into 6-well culture plates (Sarstedt, Nümbrecht, Germany) and maintained in complete MSC culture medium.

The complete MSC medium consisted of α-MEM supplemented with 10% fetal calf serum, 100 IU/mL penicillin, 50 μg/mL streptomycin, 2.5 μg/mL amphotericin B (Thermo Fisher Scientific, Dreieich, Germany), 1% sodium pyruvate, and 100 µM L-ascorbate-2-phosphate (Sigma-Aldrich, Darmstadt, Germany). After 4 days, non-adherent cells were carefully collected, avoiding disturbance of adherent tissue fragments, and the culture medium was replaced with fresh medium. The procedure was repeated twice weekly in the complete culture medium without amphotericin B.

When the cultures reached approximately 80% confluence (usually after two to three weeks), cells derived from single suspended adherent cells and those outgrowing from tissue fragments were detached using 0.02% trypsin/0.02% Na-EDTA in PBS (Sigma-Aldrich, Darmstadt, Germany). The harvested cells were then washed with culture medium and reseeded into culture dishes at 5000 cells/cm^2^. To establish bulk-cultured PDLSCs, the cell suspensions were transferred into 25 cm^2^ tissue culture flasks containing complete α-MEM medium without amphotericin B and were maintained for subsequent experiments.

We established six primary PDLSC cultures (D1–D6), each from a separate donor. All six cultures were used for phenotypic characterization and assessment of multilineage differentiation potential. Subsequent experiments were performed at passages P4–P5 to minimize passage-related variability. Not all cultures were included in every assay because sufficient cell numbers were not always available within this passage window. The choice of PDLSC cultures in subsequent experiments was not predetermined based on phenotype or osteogenic potential. Their inclusion was based solely on cell availability.

Cell morphology was assessed throughout primary culture and subsequent passages. Established cultures exhibited spindle-shaped, fibroblast-like morphology typical of mesenchymal stromal cells. Clonogenic potential was evaluated using a colony-forming unit-fibroblast assay (CFU-F). Cells were seeded at low density and cultured until discrete colonies were visible. Colonies were then fixed, stained with hematoxylin and eosin, and counted.

### 2.3. In Vitro Differentiation Potential of PDLSCs

To induce differentiation of PDLSCs into osteoblasts, cells were initially plated in six-well plates containing plastic coverslips at 6 × 10^4^ cells/well. PDLSCs were then cultured for 21 days in osteogenic medium. The osteogenic medium was complete α-MEM supplemented with 10 nM dexamethasone (Galenika, Belgrade, Serbia), 10 mM glycerophosphate, and 0.05 mM ascorbic acid (both from Sigma-Aldrich, Darmstadt, Germany). The medium was refreshed twice weekly during the culture period. After three weeks of cultivation, coverslips were rinsed with PBS and fixed in 4% paraformaldehyde for 30 min. Fixed samples were washed with distilled water and stained with 2% Alizarin Red solution (Sigma-Aldrich, Darmstadt, Germany) for 45 min. Excess dye was removed by thorough washing with distilled water. Coverslips were then mounted onto microscope slides and analyzed by light microscopy.

To induce adipogenic differentiation, PDLSCs were cultured for 21 days as described for osteogenic differentiation. The adipogenic medium was complete α-MEM supplemented with 0.5 µM dexamethasone (Galenika, Belgrade, Serbia), 0.5 µM isobutyl-methylxanthine (IBMX) (Sigma-Aldrich, Darmstadt, Germany), and 50 µM indomethacin (R&D Systems, Minneapolis, MN, USA). The culture medium was refreshed twice weekly. After three weeks, coverslips with adherent cells were fixed in 4% paraformaldehyde for 30 min, rinsed with 60% isopropanol for 5 min, and stained with 0.3% Oil Red O (Sigma-Aldrich). Thereafter, the cells were washed with tap water and stained with hematoxylin for 30–40 s. The samples were then rinsed again with tap water, mounted onto microscope slides, and analyzed under a light microscope.

Chondrogenic differentiation was carried out as previously described [18]. PDLSCs (5 × 10^5^ cells) were centrifuged at 1800 rpm for 10 min in Eppendorf tubes to form pellets. The pellets were then cultured for 28 days in chondrogenic medium (complete α-MEM with chondrogenic supplement from the Lonza Group Ltd., Basel, Switzerland). The culture medium was refreshed twice weekly. At the end of the culture period, the pellets were embedded in cryoembedding medium (Bio-Optica, Milan, Italy) and stored at −80 °C until further processing. Cryosections were prepared using a cryostat (Leica Biosystems, Barcelona, Spain), air-dried, fixed in 4% paraformaldehyde, and stained with Alcian blue solution (Sigma-Aldrich, Darmstadt, Germany) for 45 min at room temperature. In all differentiation protocols, negative controls consisted of PDLSCs maintained in complete basal MSC medium.

### 2.4. Flow Cytometry Analysis

For phenotypic characterization of PDLSC cultures, we used a large panel of monoclonal antibodies (mAbs) and staining reagents. Their names, clone designations, fluorochromes/conjugates, and manufacturers are presented in Table 1. The cells were stained with appropriate dilutions of mAbs in 2% FCS/0.01% NaN_3_/PBS for 30 min at 4 °C. After washing in PBS/0.01% NaN_3_, tubes containing biotinylated mAbs were incubated with the appropriate streptavidins for an additional 30 min. The stained and twice-washed cells were analyzed on a BD LSR II flow cytometer (BD Biosciences, Franklin Lakes, NJ, USA). Doublets were excluded based on forward scatter (FSC-A/FSC-H), and more than 5000 cells were gated according to their specific FSC-A/side-scatter (SSC)-A properties. When multiple reagents were used for simultaneous cell staining, signal overlap between channels was compensated for before each experiment using single-labeled cells. Non-specific fluorescence was determined using the appropriate isotype control antibodies. The data were analyzed offline in FlowJo v10 (BD Biosciences, Franklin Lakes, NJ, USA).

### 2.5. Irradiation Protocols

In this study, low-level laser irradiation was performed using an Epic 10 diode laser (BIOLASE, Inc., Foothill Ranch, CA, USA) operating at 940 nm with a maximum output power of 10 W. The laser was operated at 0.1 W in continuous-wave mode, and the actual output power at the sample surface was verified with a calibrated optical power meter at the beginning of the experiment. The handpiece was mounted on a stable holder with the beam axis perpendicular to the culture plate, at a fixed distance of approximately 2 cm above the cell monolayer, so that the circular laser beam (spot diameter, 7 mm; beam area, 0.385 cm^2^) fully covered the bottom of a single well of the 96-well plate. Under these conditions, the power density (irradiance) at the cell monolayer was 0.26 W/cm^2^. Cells were irradiated from above, through the culture medium (200 μL per well; medium depth, 6 mm). To minimize the effects of scattered radiation and potential cross-contamination between samples, an appropriate distance was maintained between wells containing cell cultures, and the experiment was conducted under controlled environmental conditions. Cells were exposed to laser fluences of 1 J/cm^2^, 2 J/cm^2^, 4 J/cm^2^, and 10 J/cm^2^, corresponding to exposure times of 3.8 s, 7.7 s, 15.4 s, and 38.5 s, and to delivered energies of 0.38, 0.77, 1.54, and 3.85 J per well, respectively.

For the proliferation assay, cells received a single irradiation session. For the osteogenic differentiation experiments, irradiation was performed on the day of osteogenic induction (day 0) and repeated on days 2 and 5 at half of the initial fluence (1, 2, and 5 J/cm^2^; exposure times, 3.8 s, 7.7 s, and 19.25 s), i.e., three irradiation sessions in total. Each three-session protocol is therefore referred to as an irradiation regimen and is designated by the initial fluence applied on day 0.

Control (non-irradiated) cultures were handled in exactly the same manner as the irradiated cultures. They were removed from the incubator for the same duration and positioned under the laser apparatus in the same way, but the laser was not activated. Laser irradiation did not cause a measurable increase in culture-medium temperature, as confirmed in preliminary experiments during optimization of the irradiation parameters.

### 2.6. Cell Proliferation Assay

PDLSCs were cultured in 96-well plates at 1 × 10^4^ cells/well in either complete osteogenic medium (the same medium used for PDLSC osteogenic differentiation) or a suboptimal osteogenic medium composed of 2/3 complete osteogenic medium and 1/3 complete alpha-MEM medium. Experimental cultures, plated in triplicate, were irradiated according to the above protocol at laser fluences of 1, 2, 4, and 10 J/cm^2^. Control cultures were not irradiated. After 3 or 7 days, cells were detached from the plastic substrate with 0.25% trypsin in 0.02% EDTA. Cell number and viability were determined using the Guava ViaCount assay on a Guava easyCyte Mini flow cytometer (Guava Technologies, Inc., Hayward, CA, USA). Values are expressed as the total number of cells obtained from three pooled replicate wells for each donor-derived PDLSC culture and experimental condition. The pooled sample from each donor was considered one biological experimental unit. Four independent donor-derived PDLSC cultures were analyzed (*n* = 4), and these donor-specific values were used for statistical analysis.

### 2.7. Effect of Laser Irradiation on Osteogenic Differentiation of PDSCs

PDLSCs were cultured in 96-well plates overnight at 2 × 10^4^ cells/well in complete alpha-MEM. The next day, cells were cultured in either complete osteogenic or suboptimal osteogenic medium for up to 21 days. Experimental cultures were irradiated according to the above protocol at laser fluences of 2, 4, and 10 J/cm^2^. Control cultures were not irradiated. The irradiation procedure was repeated on days 2 and 5 at half the laser fluence of the initial irradiation (1 J/cm^2^, 2 J/cm^2^, and 5 J/cm^2^, respectively). As previously stated, each repeated exposure protocol is hereafter referred to as an irradiation regimen defined by its initial fluence. For mRNA expression, cells were collected on days 3, 7, and 15. Within each donor-derived PDLSC culture, cells from 12 technical replicate wells were pooled separately for each irradiation condition and its corresponding control to ensure a sufficient number of cells for downstream analysis. Cells from different donors were not pooled. Four independent donor-derived PDLSC cultures were analyzed (*n* = 4), and each pooled donor-specific sample was considered one biological unit for statistical analysis.

### 2.8. Quantification of Osteogenic Differentiation by Alizarin Red S Staining

After 21 days of osteogenic induction, extracellular matrix mineralization was assessed by Alizarin Red S staining. Cultures grown in 96-well plates were examined under an inverted microscope (Olympus CKX41, Hamburg, Germany). The entire surface of each well was systematically scanned and imaged using a microscope-mounted digital camera. Individual images were stitched to generate a composite image of the entire culture area. Alizarin Red-positive staining was quantified by digital image analysis and expressed as the number of positive pixels per square centimeter of the culture area (pixels/cm^2^). Positive-pixel quantification was performed using an automated thresholding procedure with parameters optimized before analysis and subsequently applied consistently to all images. An investigator blinded to the treatment groups performed the image analysis.

For each PDLSC line, experiments were performed in triplicate. The mean value of the three technical replicates was calculated for each cell line and experimental condition, and these cell line-specific mean values from four independent PDLSC lines were used as the independent observations (*n* = 4) in the statistical analysis.

### 2.9. Real-Time Quantitative PCR

Gene expression was assessed in non-irradiated and laser-irradiated PDLSCs cultured under suboptimal or optimal osteoinductive conditions, as described above. Cells were collected at 3, 7, and 15 days post-osteoinduction. Total RNA was extracted from cells using the Total RNA Purification Mini Spin Kit (Genaxxon Bioscience GmbH, Ulm, Germany) according to the manufacturer’s protocol. Reverse transcription of the isolated RNA (0.1 µg) was performed using the High-Capacity cDNA Reverse Transcription Kit (Thermo Fisher Scientific, Dreieich, Germany). The synthesized cDNA was analyzed by quantitative real-time PCR (qPCR) using the FastGene^®^ 2x IC Green Universal (NIPPON Genetics, Düren, Germany) and a LineGene 9600 Plus Real-Time PCR system (Hangzhou Bioer Technology, Hangzhou, China) under the following cycling conditions: initial activation at 95 °C for 2 min, followed by 40 cycles of denaturation at 95 °C for 5 s and annealing/elongation at 60 °C for 30 s. Gene expression levels were normalized to β-actin (ACTB) and calculated using the 2^−ΔΔCt^ method [19]. The stability of ACTB expression under our experimental conditions was assessed in an initial screening experiment. As no relevant treatment-dependent variation in ACTB expression was observed, β-actin was subsequently used as the reference gene throughout the study without repeating the stability assessment in every individual donor-derived culture. For each cell line, the expression of the target gene in the analyzed samples was determined relative to that in the corresponding untreated control (cells grown in α-MEM medium without osteoinductive supplements), which was assigned a value of 1. The results are presented as fold changes, indicating the relative increase or decrease in gene expression compared with the control. Gene expression was analyzed in technical duplicate for each of four independent PDLSC cultures (*n* = 4 biological replicates). The two technical measurements for each culture were averaged to obtain a single value for that biological replicate, and these donor-specific values were used for statistical analysis. All primers (listed in Table 2) were purchased from Thermo Fisher Scientific.

### 2.10. Quantification of Soluble Osteogenic Factors in PDLSC Cultures

PDLSCs were cultured in complete osteogenic medium to induce osteogenic differentiation. Experimental cultures were irradiated with a 940 nm laser at a fluence of 2 J/cm^2^, as described previously, whereas control cultures were maintained under the same conditions without laser treatment. Cell culture supernatants from three different donor-derived PDLSC cultures (*n* = 3) were collected on days 3, 7, and 15 after osteogenic induction. The concentrations of bone morphogenetic protein-2 (BMP-2), osteoprotegerin (OPG), alkaline phosphatase (ALP), platelet-derived growth factor-BB (PDGF-BB), and leptin were measured simultaneously using the LEGENDplex™ Human Bone Formation Panel (5-plex) V02 (BioLegend, Inc., San Diego, CA, USA), according to the manufacturer’s instructions. The concentrations of soluble osteogenic factors were reported in pg/mL because, within each donor-derived PDLSC culture and at each time point, the number of cells recovered did not differ significantly between the control and PBM-treated conditions.

### 2.11. Statistical Analysis

Data distribution was evaluated using the Shapiro–Wilk normality test. For the proliferation assay and relative gene-expression analyses, differences between osteogenic control cultures (osteogenic induction without irradiation) and the corresponding PBM-treated cultures were analyzed by repeated-measures one-way ANOVA (RM-ANOVA) followed by Tukey’s multiple-comparison test. Because both cell proliferation and, in particular, osteogenic gene expression exhibit time-dependent response patterns, each experimental time point was analyzed separately rather than treating time as an additional repeated factor. For gene-expression experiments, expression in the untreated control cultures was normalized to 1. Differences between this normalized reference value and cultures subjected to osteogenic induction were evaluated using a one-sample *t*-test against the theoretical value of 1. For soluble osteogenic products, comparisons between osteogenic control cultures and the corresponding PBM-treated cultures derived from the same PDLSC line were performed using a two-tailed paired *t*-test. Analyses were performed separately at each experimental time point.

Four independent PDLSC cultures were used for the proliferation and gene-expression experiments. This sample size was supported by the study of Etemadi et al. [20], who reported a minimum sample size of four experimental units per group for the proliferation assay based on an a priori power analysis (α = 0.05, power = 80%, effect size = 0.98) and also used four experimental units per group for quantitative PCR analysis.

We also used a sample size of four independent PDLSC cultures for mineralization quantification. In a closely related in vitro study, de Melo et al. [21] quantified Alizarin Red-positive osteogenic differentiation as differentiated area (pixel^2^) using automated image analysis with TensorFlow and ArcGIS 10.8 and performed the experiments in triplicate (*n* = 3). Based on their published 7-day TensorFlow data comparing HANp with HANp + PBM, the reconstructed standardized effect size was large (Cohen’s *d* = 2.28). Using α = 0.05 and 80% power, this effect corresponds to an estimated sample size of approximately 4.2 experimental units per group.

For soluble osteogenic products, three independent PDLSC cultures were used. This sample size was supported by a closely related PBM study in human MSCs in which osteogenic factors were quantified in triplicate. Reconstruction of the published BMP-2 data indicated a large effect size (approximately *d* = 3.3), corresponding to about three experimental units per group at α = 0.05 and 80% power [22].

To further evaluate whether the sample size was adequate for the effects observed under the specific experimental conditions of the present study, effect sizes and their 95% confidence intervals (CIs) were calculated for individual experimental comparisons. Effect-size estimates were derived from observed differences between the respective experimental and control conditions, accounting for the paired/repeated-measures structure of the data where applicable. Effect sizes were expressed as Cohen’s *d_z_*, calculated as the mean of the paired differences divided by the standard deviation (SD) of the difference scores. Effect sizes were expressed as Cohen’s *d_z_* and interpreted using conventional benchmarks of 0.20, 0.50, and 0.80 for small, medium, and large effects, respectively, as summarized by Lakens (2013) [23]. Ninety-five percent CIs for *d_z_* were calculated using the non-central *t* distribution. These intervals reflect uncertainty in the effect-size estimate and are particularly important given the small number of independent experimental replicates [23].

Statistical analyses were performed using GraphPad Prism 8.0.0 (GraphPad Software, San Diego, CA, USA). Cohen’s *d_z_* effect sizes and their 95% CIs were calculated in Python 3.13.5 using SciPy 1.17.0. We used these estimates as complementary measures of the magnitude and precision of the observed effects and interpreted them alongside the corresponding *p*-values. Data are presented as mean ± SD. A *p*-value < 0.05 was considered statistically significant.

## 3. Results

### 3.1. Characterization of Primary Human PDLSCs

**Figure 1:**
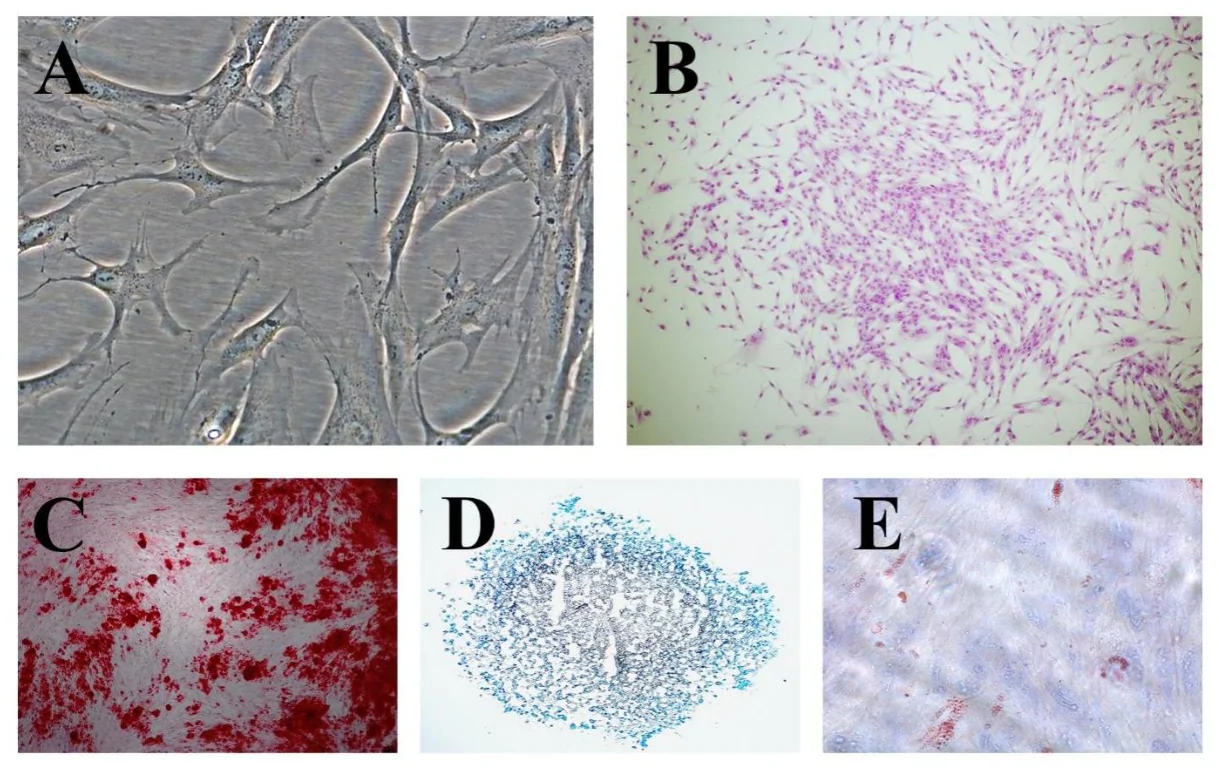
Morphological and multilineage differentiation characteristics of human periodontal ligament stem cells (PDLSCs). (**A**) Representative fibroblast-like, spindle-shaped morphology of adherent PDLSCs. (**B**) Colony-forming capacity of PDLSCs. (**C**) Osteogenic differentiation demonstrated by Alizarin Red S staining of mineralized extracellular matrix. (**D**) Chondrogenic differentiation demonstrated by Alcian blue staining of the chondrogenic pellet. (**E**) Adipogenic differentiation demonstrated by Oil Red O staining of intracellular lipid droplets. Original magnification: (**A**) ×400, (**B**) ×100, (**C**) ×200, (**D**) ×100, and (**E**) ×200.

Six primary human PDLSC cultures (D1–D6) were established as described in the Section 2. The cells exhibited typical fibroblastoid morphology, adherence to plastic, and colony-forming capability (Figure 1). PDLSC cultures demonstrated high potential for osteogenesis, moderate potential for chondrogenesis, and very low or undetectable potential for adipogenesis (Figure 1).

**Figure 2:**
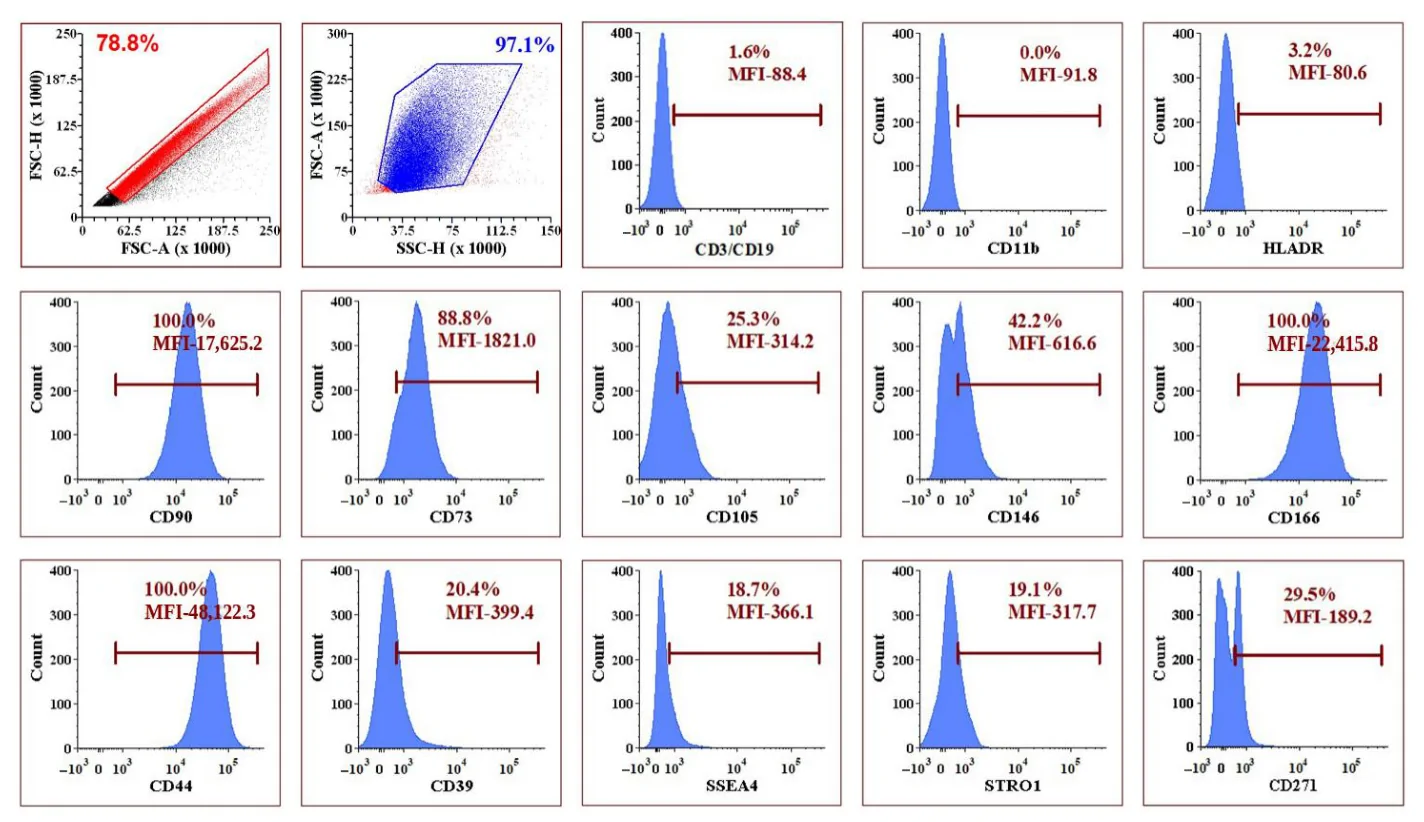
Flow cytometric profiles of the D1 PDLSC culture. Results are shown as percentages of positive cells and as mean fluorescence intensity (MFI). The first two profiles (upper row) represent the analyzed population of singlets and side scatter (SSC-H) versus forward scatter (FSC-A), respectively.

Phenotypic characteristics, shown in Table 3 and Figure 2, demonstrate high expression of CD90, CD73, CD44, and CD166. Other markers showed variable expression across the lines, including CD146 (8.2–46.2%), CD105 (13.8–25.3%), CD39 (8.1–34.8%), and CD271 (0.8–29.5%). Expression of stem-related markers was also variable, including SSEA4 (2.2–18.7%) and STRO1 (11.3–19.1%). Pericyte markers showed low expression in the two lines tested: NG2 (4.8% and 6.4%) and PDGFR (6.6% and 8.9%). The lines did not express T- and B-cell markers (CD3/CD19) or the myeloid marker CD11b. HLA-DR expression was very low (1.4–3.2%).

### 3.2. Effects of Photobiomodulation on the Proliferation of PDLSCs

**Figure 3:**
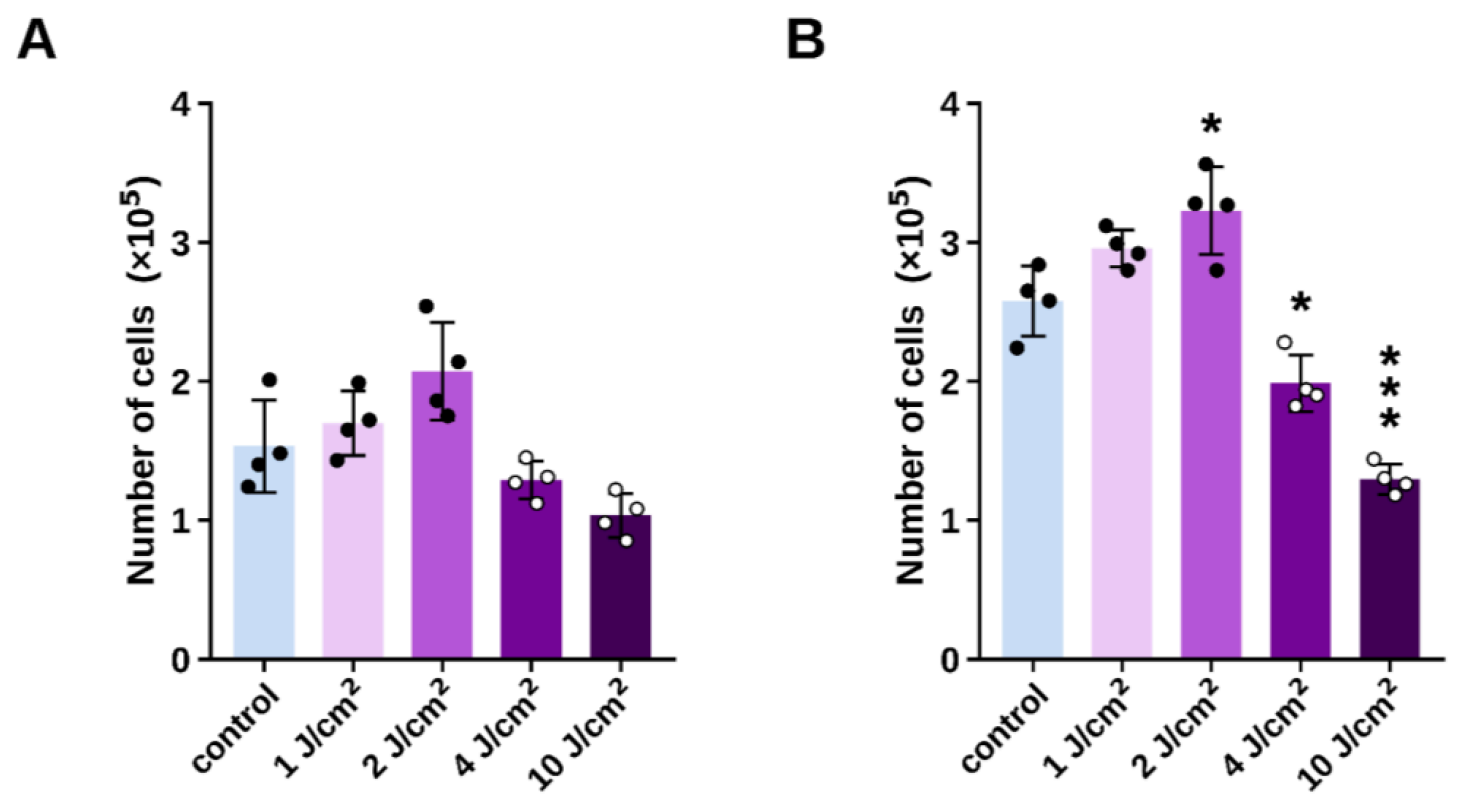
Effect of photobiomodulation on the proliferation of PDLSCs. PDLSCs (cell cultures D1, D2, D3, and D5) were treated with a laser at various fluences, as described in Section 2. Cell proliferation was indirectly assessed on days 3 (**A**) and 7 (**B**) by comparing cell numbers in laser-treated cultures with those in control, non-irradiated cultures. Values are shown as mean ± SD (*n* = 4 different donor cultures). * *p* < 0.05; *** *p* < 0.005 compared with control values (one-way RM-ANOVA followed by Tukey’s multiple-comparisons test). Dots represent values from independent donor-derived PDLSC cultures.

The modulatory effect of PBM was first examined using a proliferation assay. PDLSCs were exposed to a laser at energy densities of 1, 2, 4, and 10 J/cm^2^. Cell proliferation was measured by counting cells on days 3 and 7 after irradiation and compared with control, non-irradiated cultures as described in Section 2. Results are shown in Figure 3. Laser treatment did not significantly affect cell proliferation on day 3. However, on day 7, a fluence of 2 J/cm^2^ significantly increased cell proliferation by 25.2% compared with the control (*p* = 0.0111), whereas 4 J/cm^2^ and 10 J/cm^2^ significantly reduced proliferation by 23% and 49.8%, respectively (*p* = 0.0206 and *p* = 0.0003, respectively).

At day 7, PBM at 2 J/cm^2^ significantly increased PDLSC proliferation by 25.2% compared with the non-irradiated control (*p* = 0.0111), whereas 4 and 10 J/cm^2^ reduced proliferation by 23.0% (*p* = 0.0206) and 49.8% (*p* = 0.00003), respectively. All statistically significant changes were associated with large effect sizes according to conventional Cohen benchmarks, ranging from |*d_z_*| = 1.35 for 2 J/cm^2^ to |*d_z_*| = 3.60 for 10 J/cm^2^. The corresponding 95% CIs were relatively wide, particularly for the 2 and 4 J/cm^2^ comparisons, whereas the interval for 10 J/cm^2^ remained clearly separated from zero (Supplementary Table S1).

### 3.3. The Effect of Photobiomodulation Therapy on the Osteogenic Potential of PDLSCs

**Figure 4:**
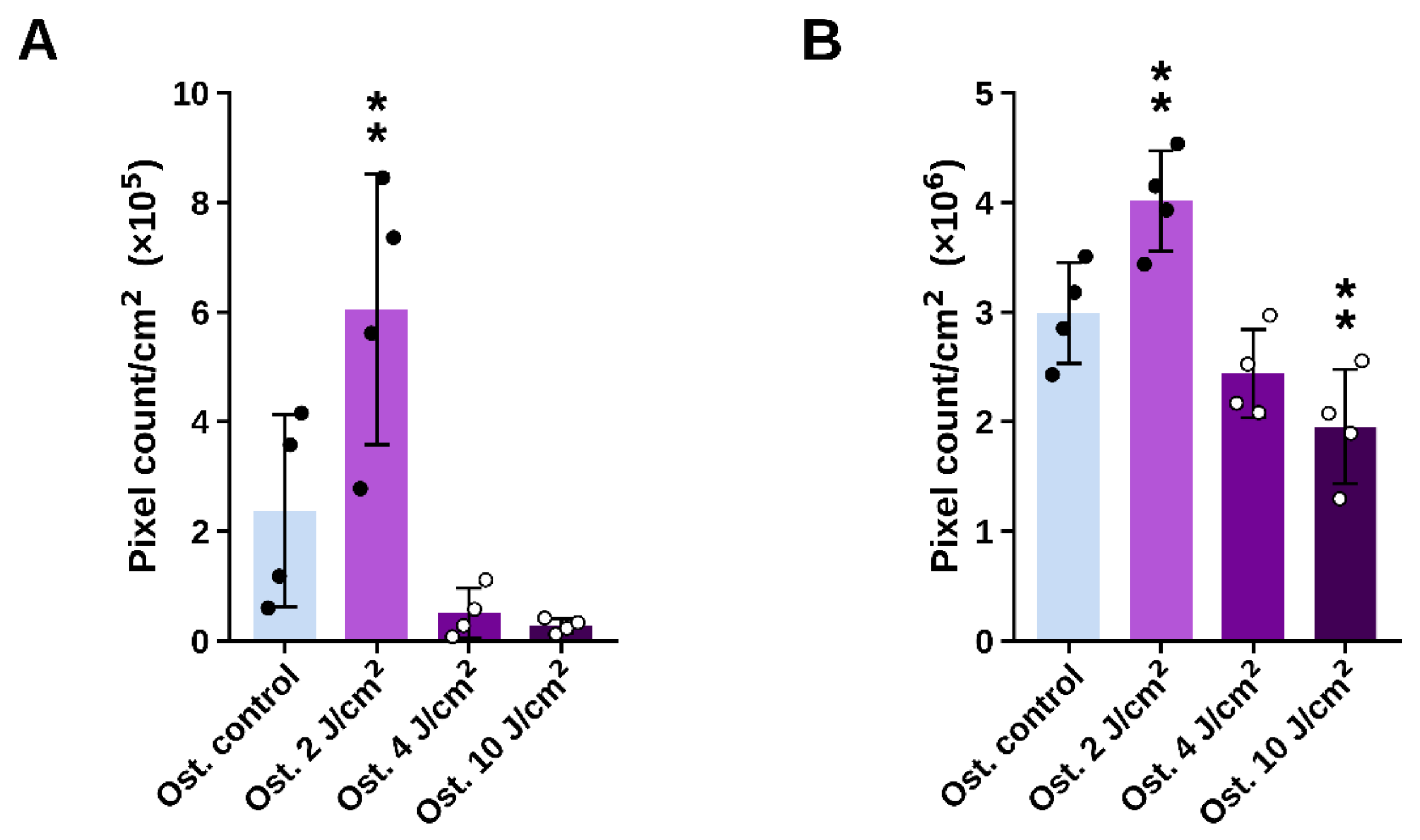
The effect of laser irradiation on osteoblastogenesis in PDLSC cultures grown in either suboptimal (**A**) or complete osteogenic (**B**) medium. PDLSCs (cell cultures D1, D2, D3, and D5) were exposed to laser irradiation at various energy densities, as described in Section 2. Results are shown as red color intensity, measured as pixel count per square centimeter (mean ± SD; *n* = 4 different PDLSC cultures). ** *p* < 0.01, compared with control values (one-way RM-ANOVA followed by Tukey’s multiple-comparisons test). Dots represent values from independent donor-derived PDLSC cultures.

**Figure 5:**
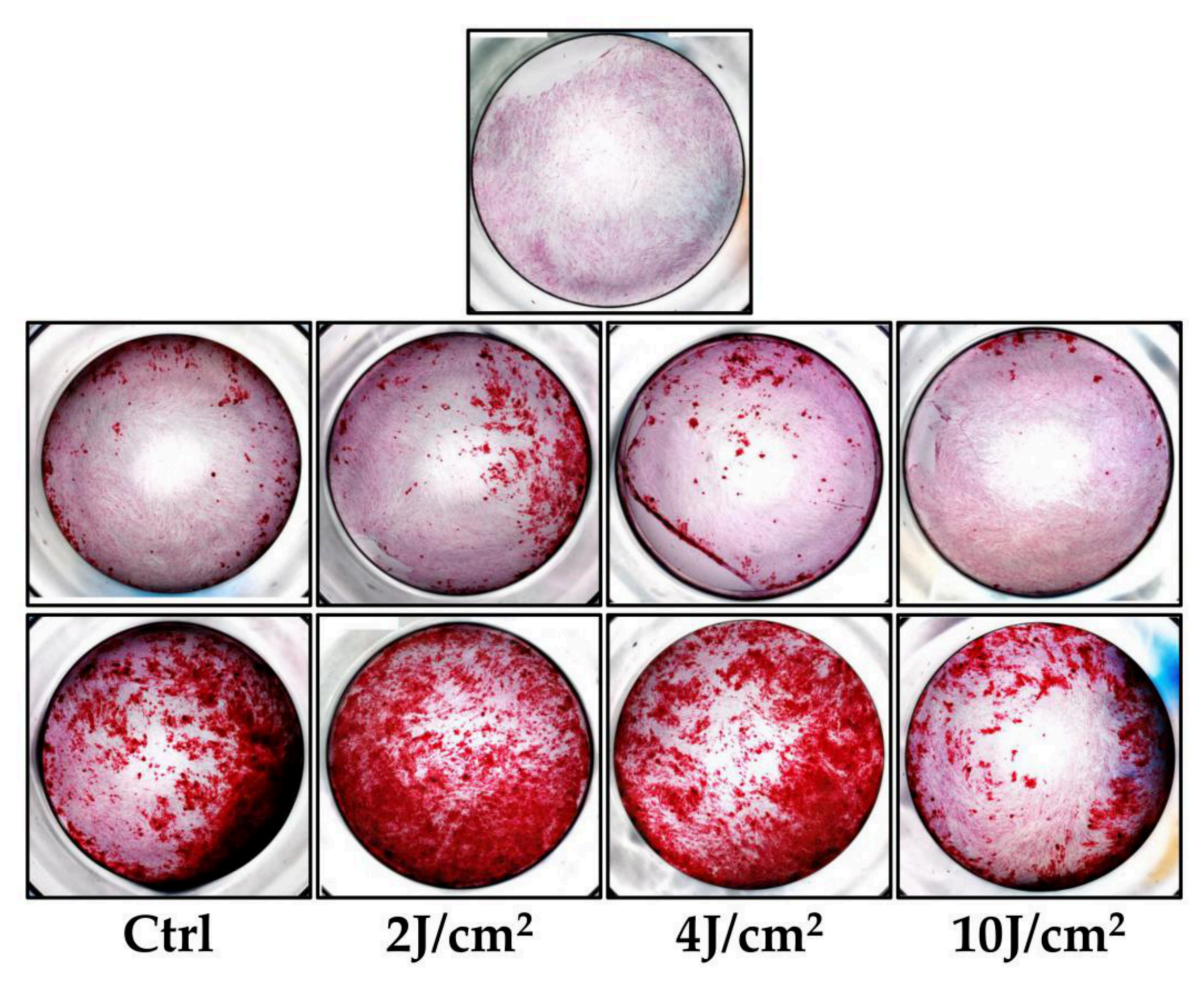
Representative Alizarin Red S-stained whole-well images showing the effects of photobiomodulation on osteogenic differentiation of human PDLSCs. The upper image shows cells cultured in basal medium without osteogenic induction. The middle and bottom rows show cells cultured in suboptimal and complete osteogenic media, respectively, without PBM treatment (Ctrl) or after irradiation with 2, 4, or 10 J/cm^2^ regimens. Red staining indicates mineralized extracellular matrix deposits.

Based on the previous results, we applied three laser fluence regimens (2 J/cm^2^, 4 J/cm^2^, and 10 J/cm^2^) and followed the irradiation protocol detailed in Section 2. Cell cultures were induced to undergo osteoblastogenesis using both suboptimal (two-thirds osteogenic medium) and complete osteogenic medium. The osteogenic potential of four PDLSC cultures (D1, D2, D3, and D5) was assessed on day 21 with Alizarin Red staining. The results are presented in Figure 4 and Figure 5.

When cells were cultured in the suboptimal osteogenic medium (Figure 4A), osteoblastogenesis was relatively low. However, irradiation at a 2 J/cm^2^ regimen resulted in a significant increase in osteogenic mineralization by 154.4% compared with the control (*p* = 0.00912), whereas the effects observed at 4 J/cm^2^ and 10 J/cm^2^ regimens were lower but did not reach statistical significance. This significant stimulatory effect was associated with a large effect size (Cohen’s *d_z_* = 3.01, 95% CI 0.54–5.48) (Supplementary Table S2).

Osteoblastogenesis was about 15 times higher in the complete osteogenic medium (Figure 4B). Under these conditions, irradiation at 2 J/cm^2^ irradiation regimen further increased osteogenic mineralization by 34.2% compared with the non-irradiated control (*p* = 0.0054), whereas 10 J/cm^2^ reduced mineralization by 34.6% (*p* = 0.0050). Both significant effects were associated with large effect sizes, with *d_z_* = 4.56 (95% CI 1.04–8.17) for 2 J/cm^2^ and *d_z_* = −2.00 (95% CI −3.78 to −0.17) for 10 J/cm^2^. The corresponding 95% CIs were relatively wide, reflecting limited precision of the effect-size estimates (Supplementary Table S2). Figure 5 illustrates representative images of these experiments.

### 3.4. The Effect of Photobiomodulation Therapy on the Expression of Osteoblastic Genes

**Figure 6:**
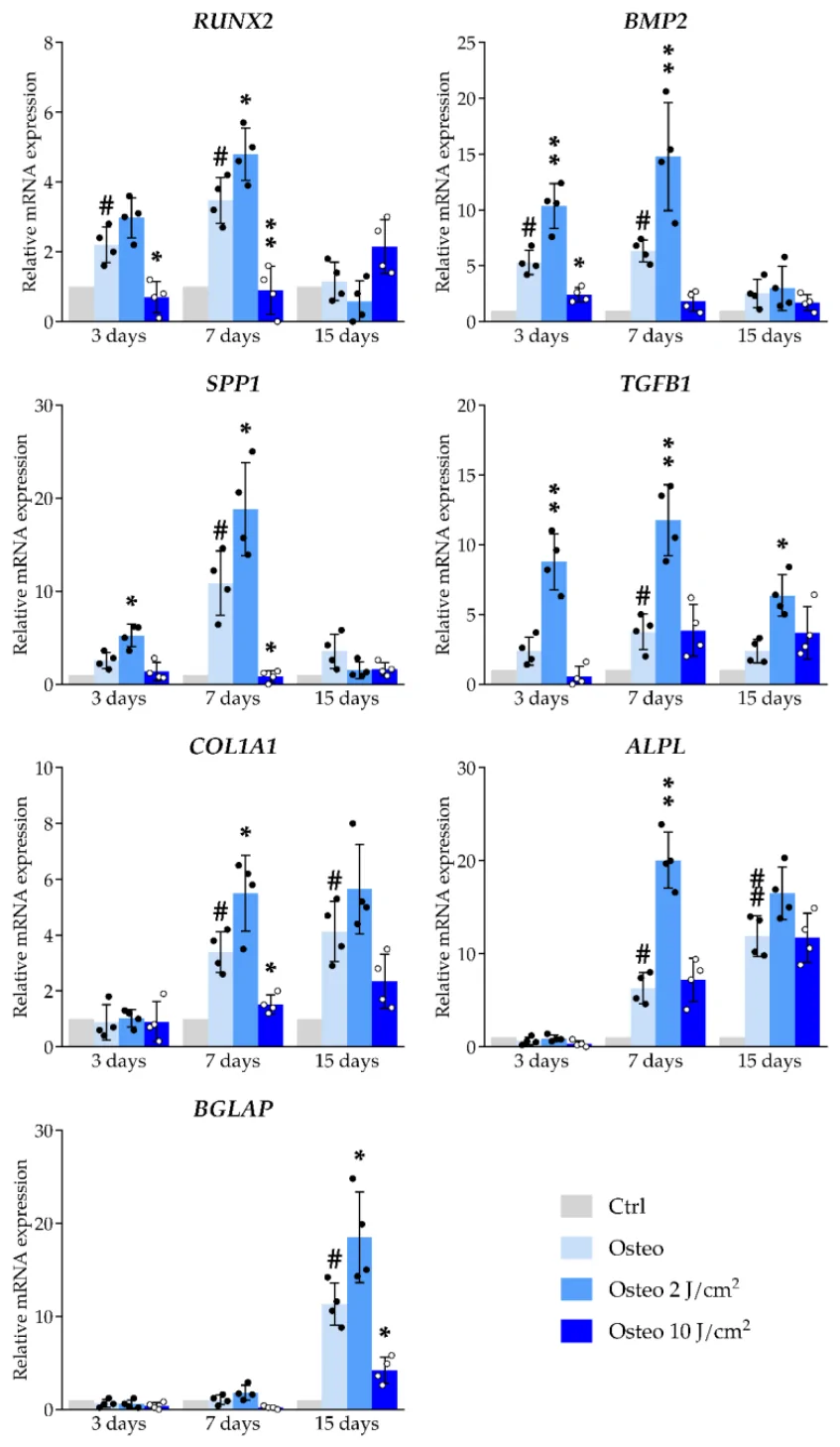
The effect of photobiomodulation therapy on the expression of osteogenic genes. Values are presented as the mean ± SD of relative mRNA expression, with negative controls standardized to 1.0 (*n* = 4 different donor cultures). # *p* < 0.05; ## *p* < 0.01 compared with negative controls (one-sample *t*-test); * *p* < 0.05; ** *p* < 0.01 compared with osteoblast (osteo) controls (one-way RM-ANOVA followed by Tukey’s multiple-comparisons test). Dots represent values from independent donor-derived PDLSC cultures. *RUNX2*, runt-related transcription factor 2; *BMP2*, bone morphogenetic protein 2; *SPP1*, secreted phosphoprotein 1; *TGFB1*, transforming growth factor beta 1; *COL1A1*, collagen type I alpha 1 chain; *ALPL*, alkaline phosphatase, biomineralization associated; *BGLAP*, bone gamma-carboxyglutamate protein.

The effects of laser irradiation on the expression of early osteoblastic genes (*RUNX2*, *BMP2*, *SPP1*, and *TGFB1*) and late osteoblastic genes (*COL1A1*, *ALPL*, and *BGLAP*) are shown in Figure 6, which presents their relative mRNA levels. Based on the previous experiments, PDLSCs (D1, D2, D5, and D6 primary PDLSC cultures) were cultured in complete osteogenic medium. Experimental cultures were laser-treated at fluences of 2 J/cm^2^ or 10 J/cm^2^ regimens, as described in Section 2. Gene expression was analyzed on days 3, 7, and 15 after osteoblastic induction. Results were expressed as relative mRNA levels compared with control cells cultured in basal medium without osteoblastic supplements.

Osteoinduction increased the expression of *RUNX2* and *BMP2* on days 3 and 7, whereas an increase in *SPP1* and *TGFB1* expression was observed on day 7. Irradiation at 2 J/cm^2^ increased *BMP2* expression by 95% on day 3 (*p* = 0.00316) and 134% on day 7 (*p* = 0.00571). *SPP1* expression increased by 105% on day 3 (*p* = 0.0472) and 73% on day 7 (*p* = 0.0403). The expression of *TGFB1* was increased at all time points, by 270% on day 3 (*p* = 0.00354) and 213% on day 7 (*p* = 0.00685), and by 167% on day 15 (*p* = 0.0171), compared with non-treated cultures. *RUNX2* expression was increased only on day 7, by 38% (*p* = 0.0448). A higher fluence regimen of 10 J/cm^2^ reduced *RUNX2* expression by 68% on day 3 (*p* = 0.0256) and 74% on day 7 (*p* = 0.00208). *BMP2* expression decreased by 55% on day 3 (*p* = 0.0400), while the decrease on day 7 (71%) did not reach statistical significance. No change in *TGFB1* expression was observed.

Osteoinduction increased the expression of *COL1A1* and *ALPL* on days 7 and 15, whereas *BGLAP* expression increased on day 15. A lower fluence of 2 J/cm^2^ increased *COL1A1* expression by 62% on day 7 (*p* = 0.0199) and *ALPL* expression by 64% on day 15 (*p* = 0.0237). In contrast, irradiation at 10 J/cm^2^ regimen reduced *COL1A1* expression by 55% on day 7 (*p* = 0.0321) and *BGLAP* expression by 63% on day 15 (*p* = 0.0256). Other differences were not statistically significant.

All statistically significant PBM-induced changes in osteoblast-related gene expression were associated with large effect sizes (|Cohen’s *d_z_*| ≥ 0.80). Among the significant comparisons, the smallest effect size was observed for *SPP1* at day 7 following 2 J/cm^2^ PBM (*d_z_* = 1.24), whereas the largest was observed for *COL1A1* at day 7 following 10 J/cm^2^ PBM (*d_z_* = −3.76; |*d_z_*| = 3.76). Several corresponding 95% CIs were relatively wide, including comparisons with *p*-values close to the 0.05 significance threshold (Supplementary Table S3).

### 3.5. The Effect of Photobiomodulation Therapy on the Production of Secreted Biomolecules in Osteoblastic Cultures

**Figure 7:**
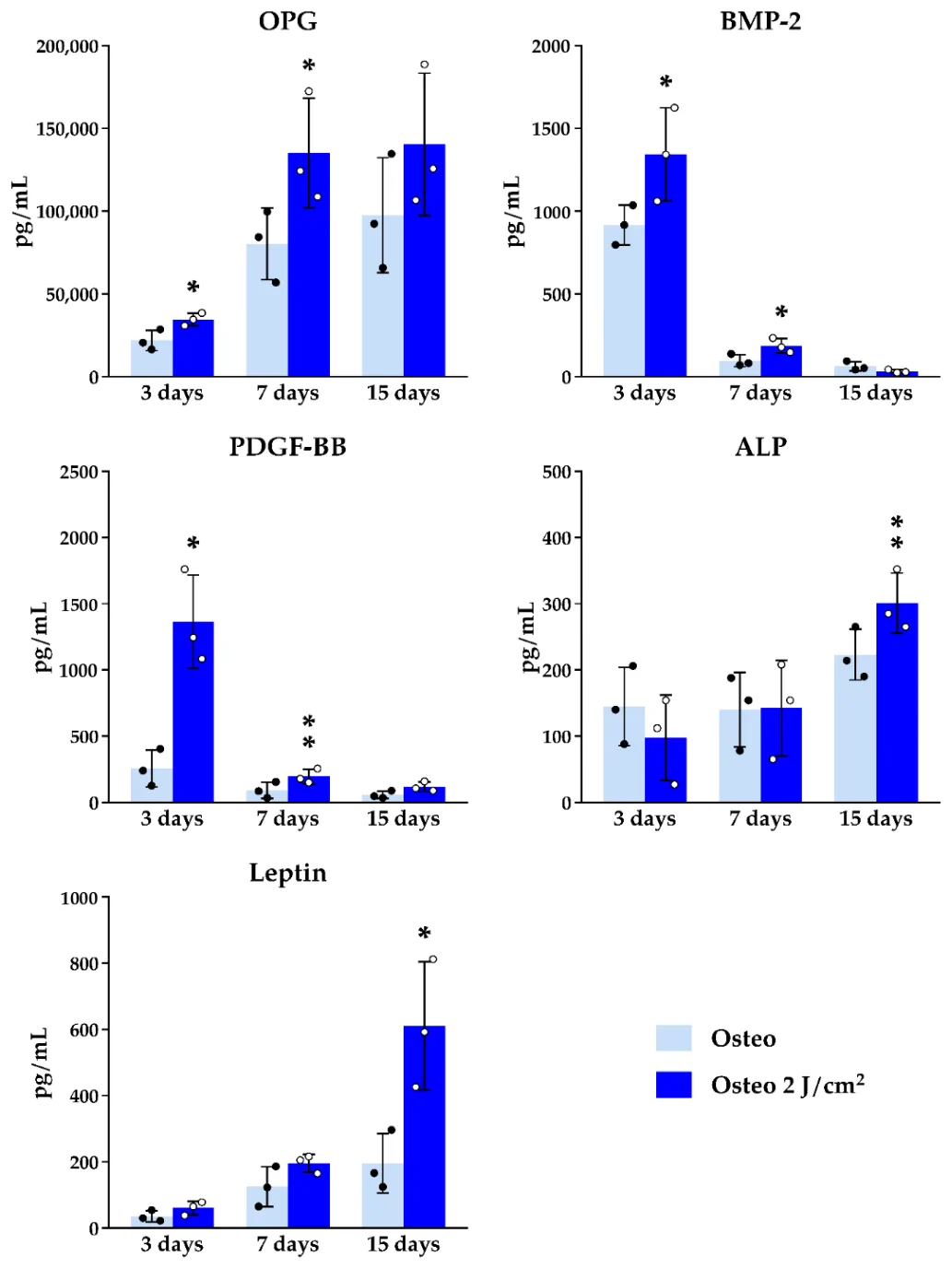
The effect of photobiomodulation therapy on the secretion of osteogenesis-related biomolecules in PDLSC cultures. Values are presented as the mean ± SD (*n* = 3 different donor-derived PDLSC cultures) (D1, D2, and D5). * *p* < 0.05; ** *p* < 0.01, compared with corresponding controls (osteoblastic cultures without laser treatment) (two-tailed paired *t*-test). Dots represent values from independent donor-derived PDLSC cultures. BMP-2, bone morphogenetic protein 2; OPG, osteoprotegerin; ALP, alkaline phosphatase; PDGF-BB, platelet-derived growth factor-BB.

Five biomolecules (BMP-2, OPG, ALP, PDGF-BB, and Leptin) in the culture supernatants of PDLSCs stimulated toward osteoblastogenic differentiation were analyzed. Experimental cultures were treated with a stimulatory fluence regimen (2 J/cm^2^). Results are presented in Figure 7. PBM therapy stimulated the production of all biomolecules, but the effect was clearly time-dependent. The levels of BMP-2 and PDGF-BB peaked on day 3, and laser treatment further increased their concentrations in the supernatants (*p* = 0.0450 and *p* = 0.0449, respectively), resulting in increases of 46.7% and 431.0%, respectively. On day 7, PBM increased the production of these biomolecules by 93.3% and 114.3%, respectively (*p* = 0.0190 and *p* = 0.0046, respectively). OPG production peaked on days 7 and 15. However, the stimulatory effect of PBM was statistically significant on days 3 and 7, with increases of 58.3% and 63.8%, respectively (*p* = 0.0414 and *p* = 0.0289, respectively). The stimulatory effect of PBM on ALP and leptin production was most pronounced on day 15, with increases of 34.8% and 212.3%, respectively (*p* = 0.0038 and *p* = 0.0341, respectively).

All statistically significant PBM-induced changes in soluble osteogenic mediators were likewise associated with large effect sizes (|Cohen’s *d_z_*| ≥ 0.80). The smallest significant effect size was *d_z_* = 2.63, observed for both BMP-2 and PDGF-BB at day 3, whereas the largest was observed for ALP at day 15 (*d_z_* = 9.33). Several corresponding 95% confidence intervals were relatively wide, including comparisons with *p*-values close to the 0.05 significance threshold (Supplementary Table S4).

## 4. Discussion

This study aimed to investigate the effects of different 940 nm laser irradiation fluences on PDLSC proliferation and their osteogenic differentiation. For this purpose, we established six primary human PDLSC cultures that fulfilled the basic characteristics of MSCs, consistent with the minimal criteria proposed by Dominici et al. [24]. A high proportion of cells was positive for CD90, CD73, CD166, and CD44. CD105 expression was more variable among the cells. Only a fraction of PDLSCs expressed typical stem/progenitor markers, including STRO-1, SSEA-4, and CD146. These findings largely agree with previous publications [24,25]. It is believed that subpopulations expressing STRO-1/CD146, CD105, or CD271 can possess greater clonogenic and osteogenic capacity [26,27]. However, these subsets are not equally represented across all our PDLSC cultures. A minor subset was also positive for pericyte markers, such as NG2 and PDGFR. These findings are consistent with the pericyte origin of MSCs or their subsets, including PDLSCs [28].

The heterogeneity of PDLSCs is well known and important for interpreting functional assays. Routine isolation and culture procedures do not yield a pure population of PDLSCs. These cells are initially recovered as part of a heterogeneous periodontal ligament cell population enriched in fibroblast-like cells [29]. Fibroblasts are the predominant resident cells of the periodontal ligament. Their principal functions are related to synthesis and remodeling of the extracellular matrix, particularly collagen, as well as maintenance of tissue integrity and adaptation to mechanical forces [30]. In contrast, PDLSCs display a higher clonogenic potential and a more pronounced capacity for self-renewal. Owing to their multilineage differentiation potential, they can contribute to the formation of several components of the periodontal attachment apparatus, including bone-forming cells, fibroblasts, and cementoblasts [29,30]. Therefore, primary PDLSC cultures should be regarded as heterogeneous populations of periodontal stromal cells. They may contain variable proportions of true progenitor cells and more differentiated fibroblast-like cells.

Our six PDLSC cultures showed a pronounced capacity for osteoblastic differentiation and a moderate potential for chondrogenic differentiation. In contrast, adipogenic differentiation of these cells was weak or absent in some lines. The observed differentiation process is in line with a predominant commitment of periodontal ligament progenitor cells toward osteogenic and cementogenic lineages [24,25]. Therefore, the low adipogenic response of PDLSCs may partly reflect these intrinsic lineage differentiation pathways. This phenomenon may also depend on the cellular composition of individual PDLSC cultures. In particular, cultures enriched in periodontal ligament fibroblasts are likely to exhibit weaker adipogenic differentiation. Accordingly, differences in adipogenesis observed among PDLSC cultures may be related to variations in the relative proportion of stem/progenitor cells and fibroblast-like cells within individual isolates.

To characterize osteogenic differentiation, we analyzed *RUNX2*, *BMP2*, *SPP1*, *TGFB1*, *COL1A1*, *ALPL*, and *BGLAP*, which collectively reflect osteoblast commitment, matrix formation, and mineralization. *RUNX2*-centered transcriptional programs and BMP/TGF-β signaling coordinate early osteogenic commitment, whereas *ALPL*, *COL1A1*, *SPP1*, and *BGLAP*/osteocalcin broadly reflect subsequent matrix maturation and mineralization [31,32]. In our cultures, the temporal expression pattern followed this sequence: earlier changes in *RUNX2*, *BMP2*, *SPP1*, and *TGFB1*, followed by later increases in *COL1A1*, *ALPL*, and *BGLAP*. BMP-2 and soluble ALP generally paralleled the corresponding gene-expression profiles [31,32].

Given the conflicting data on the biological efficacy of 940 nm irradiation in PDLSCs, we specifically investigated the effects of a 940 nm diode laser on their osteogenic response. Our results support the assumption that this wavelength can modulate PDLSC proliferation and osteoblastic differentiation. However, the effect was clearly fluence-dependent. The lower fluence of 2 J/cm^2^ promoted proliferation, mineralized matrix formation, and the expression of all osteogenic markers we investigated. In addition, we detected, for the first time, increased levels of soluble factors from differentiating PDLSCs, including OPG, PDGF-BB, and leptin. We observed the stimulatory effect under both suboptimal and complete osteogenic conditions. In contrast, the inhibitory effect of the 10 J/cm^2^ regimen became evident only under complete osteogenic induction. Under these conditions, 10 J/cm^2^ reduced mineralization and downregulated several key osteoblast-related genes, but not all. These findings are consistent with the broader concept that PBM effects are critically dependent on dose parameters [14,20,33,34].

The available literature on in vitro studies collectively indicates that PBM can modulate PDLSC behavior, but the biological response depends strongly on wavelength, fluence, irradiation schedule, and the functional status of these cells [14,20,33,34,35]. The earliest study by Soares et al. examined the use of an InGaAlP diode laser at 660 nm. Other characteristics were as follows: output power of 30 mW; fluences (energy densities) of 0.5 and 1.0 J/cm^2^; irradiation schedule: 0 and 48 h. The authors showed that irradiation at 1.0 J/cm^2^ increased hPDLSC proliferation and mitochondrial activity, as assessed by Trypan blue exclusion and the MTT assay. A fluence of 0.5 J/cm^2^ was less effective [36]. These early findings were extended by Rigi-Ladez et al., who compared 810- and 940 nm near-infrared diode lasers at 0.5, 1.5, and 2.5 J/cm^2^ (100 mW) and found that 940 nm at 2.5 J/cm^2^ produced the greatest increases in viability and proliferation, with no evidence of apoptosis [33]. Our results are consistent with these findings, showing that 940 nm laser irradiation at 2 J/cm^2^ stimulated PDLSC proliferation after 7 days of prolonged cultivation. Thus, our data and previous 940 nm studies [33] suggest that the apparently unfavorable profile of this wavelength reported by Mylona et al. [14] may reflect protocol variability rather than the wavelength itself.

More relevant evidence for the present work comes from the literature showing that PBM promotes osteogenic differentiation and matrix mineralization in PDLSCs. Yamauchi et al. used a 650 nm red LED and screened irradiation fluences from 0 to 10 J/cm^2^, identifying 8 J/cm^2^ as the most effective parameter. The authors evaluated PDLSC osteogenic differentiation using an extensive panel of markers and demonstrated that the stimulatory effect of laser irradiation depended on extracellular signal-regulated kinase 1/2 (ERK1/2) signaling [37]. Gholami et al. also used a 940 nm diode laser at 100 mW in continuous-wave mode, applying 4 J/cm2 over three sessions at 48 h intervals. They observed that cell growth, as determined by MTT, was not significantly altered. However, mineralized tissue deposition increased with increased expression of vascular endothelial growth factor (VEGF) and BMP [38]. More recently, Etemadi et al. showed that one versus two irradiation sessions using 635- and 980 nm lasers, alone or in combination, produced different effects on PDL mesenchymal cell viability and osteogenic differentiation, further emphasizing the importance of irradiation schedule [39]. Chaweewannakorn et al. further demonstrated that wavelength selection influences the balance between proliferation and differentiation. In this context, daily LED irradiation was performed at 630, 680, or 830 nm, all at 3.5 J/cm^2^. They showed that proliferation was better under 830 nm irradiation, and osteoblastic differentiation and mineralization were better under 630 and 680 nm irradiation as measured by MTT, ALP activity, and Alizarin Red staining [40]. Another study also showed a clear pro-osteogenic effect of red LED irradiation at fluences of 1, 3, and 5 J/cm2 under osteogenic conditions. Osteogenesis was assessed using ALP activity, Alizarin Red staining, RT-PCR, and Western blotting for multiple genes/proteins, including *RUNX2*, *OCN*, *OPN*, and *BSP*. The greatest early increase in ALP activity was reported at 5 J/cm^2^ [41].

As reported in previous PDLSC studies [36,38,39,40], repeated irradiation schedules are commonly used in experimental models of osteogenic differentiation and are typically described by the initial fluence. Accordingly, we refer to our experimental regimens in the same way, because cumulative values represent only the arithmetic sum of the radiant exposures delivered at separate time points. However, the cumulative effect of repeated exposures may contribute to the final biological response [39]. Such repeated-exposure models also reflect the general concept of clinical PBM protocols involving multiple treatment sessions [3,5], although direct dose equivalence between in vitro and clinical conditions cannot be assumed.

Abo El-Dahab and others used a 970 nm infrared diode laser at 200 mW and 4 J/cm^2^ for 3 s to study proliferation and osteogenic differentiation of human PDLSCs. They showed increased viability and proliferation, changes in the cell-cycle profile, and upregulation of osteoblastic genes (*OCT4*, *SOX2*, *NANOG*, *RUNX2*, *ALP*, *OPN*, and *OCN*) on day 14 after induction of osteoblastogenesis [34]. Our study differs from previous ones because we showed that changes in gene expression upon laser irradiation were clearly time-dependent and generally followed the dynamics of their expression during the osteoblastogenic process. Collectively, these previous studies indicate that PBM can stimulate both early osteogenic commitment and later mineralization, but that the optimal protocol may vary depending on the desired endpoint [34,35,36].

As mentioned earlier, our results clearly showed a dual effect of PBM using a 940 nm diode laser. In this context, the strongest evidence that the PDLSC response to PBM is fluence-dependent comes from the two additional studies. In the first study, an Nd: YAG laser at 1064 nm was applied at 2, 4, 6, and 8 J/cm^2^. Osteogenesis was assessed by CFU-F, MTT, ALP staining and activity, Alizarin Red S staining, the expression of typical osteoblastic genes (*RUNX2*, *ALP*, *osteocalcin*, and *BMP2*), and the production of inflammatory cytokines. PBM at 2–6 J/cm^2^ enhanced proliferation and osteogenic differentiation of PDLSCs, involving BMP/Smad signaling [42]. In contrast, irradiation with 8 J/cm^2^ significantly suppressed osteogenesis. In a second study by the same research group, Nd:YAG irradiation at fluences of 4 and 8 J/cm^2^ again showed a dose-dependent stimulatory effect on human PDLSCs isolated from the periodontal ligament of patients with periodontitis. In this context, a fluence of 16 J/cm^2^ significantly suppressed proliferation and osteogenic differentiation, followed by increased expression of oxidative and inflammatory stress markers, including ROS, malondialdehyde (MDA), and inflammatory cytokines [43]. These data are highly relevant because they directly support a biphasic dose response of 1064 nm laser irradiation, in which low-to-intermediate fluences are beneficial, whereas higher fluences become inhibitory [42,43]. Studies using inflamed PDLSCs [44] and TNF-α-stimulated inflammatory conditions [45] further support this concept.

Understanding the mechanisms underlying the different biological responses to PBM fluence is important. However, these mechanisms were not directly tested in the present study and are therefore discussed as plausible explanations rather than experimentally demonstrated pathways. Mechanistically, the observed fluence-dependent response may reflect a balance between beneficial mitochondrial/redox signaling and cellular stress. PBM with red and near-infrared light can influence mitochondrial activity, synthesis of adenosine triphosphate (ATP), mitochondrial membrane potential, transient ROS production, and intracellular Ca^2+^ signaling. At moderate levels, ROS may act as second messengers rather than mediators of oxidative damage [46]. Consistent with this, recent evidence shows that 940 nm PBM induces osteoblastic differentiation of human dental pulp stem cells with concomitant ROS production and mitochondrial membrane potential [47]. Similarly, PBM-induced human MSC osteogenic differentiation was associated with Akt activation and low ROS generation, and Akt inhibition decreased ROS generation and osteogenic response [22]. Recent systematic evidence [35] indicates that Wingless-related integration site/β-catenin (Wnt/β-catenin), BMP/Smad, and phosphoinositide 3-kinase/protein kinase B (PI3K/Akt) signaling are involved in PBM-induced osteogenesis. Stimulatory effects specifically in PDLSCs have been associated with ERK1/2 signaling [36], BMP/Smad activation [47], and transient receptor potential vanilloid 1 (TRPV1)-mediated Ca^2+^ signaling [13].

In contrast, at higher fluences, excessive redox activation may shift physiological signaling toward oxidative stress and an inhibitory cellular response [43,48]. This is consistent with findings in human PDLSCs, in which higher-fluence irradiation suppressed proliferation and osteogenic differentiation and was accompanied by increased ROS, MDA, and inflammatory cytokines [43]. Importantly, in our study, the inhibitory effect observed at the 10 J/cm^2^ fluence regimen was not accompanied by reduced cell viability or increased apoptosis, arguing against overt cytotoxicity as the explanation for impaired osteogenesis. Thus, oxidative/redox imbalance, altered mitochondrial signaling, or activation of other stress-related pathways remain plausible but unproven mechanisms.

We also quantified five soluble factors in PDLSC culture supernatants: ALP, BMP-2, OPG, PDGF-BB, and leptin. During osteogenic differentiation, changes in secreted ALP and BMP-2 levels were generally consistent with the expression profiles of their corresponding genes. We also found a similar increase in their concentrations after exposure to stimulatory fluences of a 940 nm diode laser, suggesting that PBM may affect both the transcriptional activity and the secretory profile of PDLSCs during osteogenic induction.

OPG is a soluble receptor activator of nuclear factor kappa-B ligand (RANKL) decoy receptor. OPG inhibits RANKL from binding to RANK and thus inhibits osteoclast differentiation and bone resorption [49]. The effect of PBM on OPG production by PDLSCs during osteoblastic differentiation has been poorly studied. Our finding of increased soluble OPG after PBM is supported by recent studies that showed increased OPG gene expression in PDLSCs [50].

PDGF-BB promotes proliferation and chemotaxis in MSCs [51] and may enhance proliferation, migration, and osteogenic differentiation of PDLSCs through Wnt/β-catenin signaling [52]. However, under osteoinductive conditions, PDGF-BB has also been associated with increased proliferation but reduced mineralization [51,53]. To our knowledge, PBM effects on PDGF-BB secretion by PDLSCs during osteogenic differentiation have not been specifically examined. Nevertheless, our findings are consistent with clinical evidence showing increased PDGF-BB levels in palatal wound fluid during early healing after free gingival graft harvesting [54].

Leptin, in addition to its metabolic functions, is expressed by human osteoblasts and can act locally to stimulate osteoblast activity and increase mineralization during the late phase of osteoblast differentiation [55]. Although direct evidence of leptin secretion by PDLSCs is scarce, human periodontal ligament cells have been shown to express leptin and its receptor. Recent findings suggest that leptin signaling and LepR-expressing PDLSC populations may be involved in regulating osteogenic differentiation and periodontal hard-tissue formation [56,57]. To our knowledge, PBM-induced leptin production has not yet been specifically demonstrated in osteoinduced PDLSC cultures. However, fibroblast-based wound-healing models and periodontal clinical samples suggest that PBM may modulate signaling via leptin-leptin receptor interactions as an additional mechanism of PBM action [58].

From a translational perspective, these findings emphasize the importance of fluence selection when considering PBM for periodontal or orthodontic regenerative applications. Among the conditions tested in this in vitro model, the 2 J/cm^2^ regimen produced the most consistent pro-osteogenic response, whereas higher fluences, particularly 10 J/cm^2^, were inhibitory. However, these findings should not be interpreted as defining an optimal clinical dose, because tissue optical properties, mechanical loading, inflammation, vascular and multicellular interactions may substantially modify the biological response in vivo [15].

This study has several limitations. First, as an in vitro study, it cannot fully capture the complexity of the periodontal microenvironment. Second, the study was limited to a single laser wavelength (940 nm) and a restricted range of fluences and treatment frequencies, while other relevant PBM parameters were not tested. Third, the irradiation protocol did not include experimental models that reflect clinical PBM use, such as combining laser light with mechanical loading relevant to OTM or culturing PDLSCs under inflammatory stimuli to mimic periodontitis. Fourth, secreted biomolecules were analyzed only after treatment with the stimulatory fluence regimen of 2 J/cm^2^, which was prioritized because of its consistent pro-osteogenic effect and potential relevance for regenerative applications. Therefore, these findings cannot be extrapolated to the inhibitory 10 J/cm^2^ condition, and the effects of higher fluence on the secretion of BMP-2, OPG, ALP, PDGF-BB, and leptin remain to be determined.

Fifth, although some experimental endpoints used slightly different combinations of donor-derived PDLSC cultures, all analyses were performed within the respective donor sets. Nevertheless, this should be considered when directly integrating findings across different experimental endpoints. Sixth, some statistically significant findings, particularly those with *p*-values close to 0.05, were accompanied by wide 95% CIs for the effect-size estimates and should therefore be interpreted with caution. A larger number of independent donor-derived PDLSC cultures would improve precision and strengthen the robustness of the findings. Finally, although mineralization, osteoblastic gene expression, and soluble mediators were assessed, the molecular mechanisms underlying the observed PBM effects were not directly investigated. Despite these limitations, the results obtained are valuable and provide a basis for further research.

## 5. Conclusions

The results demonstrate that PBM with a 940 nm diode laser modulates the proliferation and osteogenic differentiation of human PDLSCs in culture, and that the effect is clearly fluence-dependent. A fluence of 2 J/cm^2^ stimulated mineralization, osteoblastic gene expression, and secretion of soluble mediators relevant to osteoblastogenesis, whereas higher fluences showed inhibitory effects. These findings support the concept that fluence selection is a critical determinant of PBM efficacy. Overall, these in vitro findings suggest that 940 nm PBM may be relevant to future periodontal and orthodontic regenerative research. However, further mechanistic, preclinical, and clinical studies are required before these findings can be translated into specific therapeutic protocols.

## Figures and Tables

**Table 1:** Monoclonal antibodies and staining reagents used for flow cytometry analysis.

Antibody/Reagent Name	Clone	Fluorochrome/Conjugate	Manufacturer
anti-STRO-1	STRO-1	FITC	BioLegend
anti-CD90	5E10	PE	BioLegend
anti-CD73	AD2	Biotin	BioLegend
anti-SSEA-4	MC-813-0	Biotin	BioLegend
anti-CD166	3A6	PE	BioLegend
anti-NG2	MEL62	Purified/unconjugated	BioLegend
anti-CD105	43A3	APC	BioLegend
anti-CD271	ME20.4	FITC	BioLegend
anti-CD11b	ICRF44	FITC	BioLegend
anti-HLA-DR	L243	PE	BioLegend
anti-PDGFR	D-6	Alexa Fluor 546	Santa Cruz Biotechnol.
anti-CD146	P1H12	Alexa Fluor 488	Santa Cruz Biotechnol.
anti-CD39	eBioA1	FITC	eBioscience
anti-CD3	OKT3	APC	eBioscience
anti-CD19	HIB19	APC	eBioscience
anti-CD44	IM7	APC-Cy7	BioLegend
IgG1 isotype control	MOPC-21	FITC	BioLegend
IgG1 negative control	MOPC-21	PE	BioLegend
IgG1 negative control	MOPC-21	APC	BioLegend
IgG1 negative control	MOPC-21	APC-Cy7	BioLegend
streptavidin	N/A	APC	BioLegend
streptavidin	N/A	APC-Cy7	BioLegend

Abbreviations—APC: allophycocyanin; APC-Cy7: allophycocyanin-cyanine 7 tandem conjugate; Biotin: biotin conjugate, detected using fluorochrome-conjugated streptavidin; CD: cluster of differentiation; CD271: nerve growth factor receptor (NGFR/p75NTR/LNGFR); FITC: fluorescein isothiocyanate; HLA-DR: human leukocyte antigen-DR; NG2: neural/glial antigen 2; PDGFR: platelet-derived growth factor receptor; PE: phycoerythrin; SSEA-4: stage-specific embryonic antigen-4; STRO-1: stromal cell surface antigen-1. Manufacturers: BioLegend—Basel, Switzerland; eBioscience—San Diego, CA, USA; Santa Cruz Biotechnology—Dallas, TX, USA.

**Table 2:** Primer sequences used for quantitative real-time PCR.

Gene	Primer Direction	Sequence (5′–3′)
*β-actin*	Forward	TCAGTAACAGTCCGCCTAGAAGCA
	Reverse	TTGCTGACAGGATGCAGAAGGAGA
*COL1A1*	Forward	TCGGAGGAGAGTCAGGAAGG
	Reverse	AACAGAACAGTCTCTCCCGC
*SPP1*	Forward	TTCTGCGGCAAGAGGTTCACTC
	Reverse	GTGTTTGCTCAGGTGGTCGCTT
*BMP2*	Forward	GGGGTGGGGGAAAGGTAATG
	Reverse	TCGGGTTATCCAGGTTTTGCT
*RUNX2*	Forward	GCGGTGCAAACTTTCTCCAG
	Reverse	TCACTGTGCTGAAGAGGCTG
*BGLAP*	Forward	GACTGTGACGAGTTGGCTGA
	Reverse	CACATCCATAGGGCTGGGAG
*TGFB1*	Forward	GGACACCAACTATTGCTTCAGCTCC
	Reverse	AGGCTCCAAATGTAGGGGCAGGGCC
*ALPL*	Forward	GGGCATTGTGACTACCACTC
	Reverse	AGTCAGGTTGTTCCGATTCA

**Table 3:** Phenotypical characteristics of primary human PDLSC cultures (D1–D6).

Marker	D1 (%)	D2 (%)	D3 (%)	D4 (%)	D5 (%)	D6 (%)
Ctrl	0.6	0.7	0.4	1.1	0.6	0.9
CD90	100.0	100.0	99.8	99.9	98.9	99.0
CD146	42.2	19.6	8.2	12.3	46.2	25.8
CD105	25.3	22.9	23.6	13.8	NT	NT
STRO1	19.1	15.9	11.9	11.3	18.2	9.7
CD39	20.4	8.1	16.0	11.5	34.8	26.2
SSEA4	18.7	15.4	3.2	2.2	NT	17.4
CD73	88.8	73.4	96.8	93.4	99.0	98.6
CD166	100.0	99.9	99.8	99.9	97.6	99.4
NG2	NT	NT	NT	NT	6.4	4.8
PDGFR	NT	NT	NT	NT	8.9	6.6
CD44	100.0	100.0	99.8	99.9	99.0	99.6
CD271	29.5	10.7	0.8	8.9	19.2	NT

Results of individual cell lines are presented in %. NT—Not Tested.

## Data Availability

The data presented in this study are available upon request from the corresponding author.

## Supplementary Materials

The following supporting information can be downloaded at: https://www.mdpi.com/article/10.3390/dj14100676/s1. Table S1: Effect of photobiomodulation at different fluences on PDLSC proliferation after 3 and 7 days; Table S2: Effect of photobiomodulation at different fluences on PDLSC mineralization under suboptimal and complete osteogenic conditions; Table S3: Statistical analysis of osteoblastic gene expression (fold changes) in osteogenic cultures; Table S4: Statistical analysis of secreted biomolecules in osteoblastic cultures.
