# Supplementary material for: Fluence-Dependent Effects of 940-nm Photobiomodulation on the Proliferation and Osteoblastic Differentiation of Human Periodontal Ligament Stem Cells
Source: Dent J (Basel). 2026 Oct 16;14(10):676. doi: 10.3390/dj14100676 (PMC13703215; doi:10.3390/dj14100676)
Supplement: Supplementary file 1 [file dentistry-14-00676-s001.zip › dentistry-4517835-supplementary.pdf]

Table S1. Effect of photobiomodulation at different fluences on PDLSC proliferation after 3 and 7 days

Time	Fluence	Mean ± SD (×10^5 cells)	Min–max (×10^5 cells)	Tukey-adjusted p	Cohen's d_z (95% CI)
3 days	Control	1.53 ± 0.33	1.24–2.01	—	—
3 days	1 J/cm²	1.70 ± 0.23	1.43–1.99	0.921	1.31 (−0.13–2.66)
3 days	2 J/cm²	2.07 ± 0.35	1.75–2.54	0.119	0.84 (−0.37–1.97)
3 days	4 J/cm²	1.29 ± 0.14	1.12–1.45	0.746	−0.56 (−1.59–0.55)
3 days	10 J/cm²	1.03 ± 0.16	0.85–1.22	0.163	−1.11 (−2.36–0.23)
7 days	Control	2.58 ± 0.25	2.24–2.84	—	—
7 days	1 J/cm²	2.96 ± 0.13	2.80–3.12	0.188	1.00 (−0.28–2.20)
7 days	2 J/cm²	3.23 ± 0.32	2.80–3.56	**0.0111***	1.35 (−0.11–2.72)
7 days	4 J/cm²	1.98 ± 0.20	1.82–2.28	**0.0206***	−1.37 (−2.76–0.10)
7 days	10 J/cm²	1.30 ± 0.11	1.18–1.44	**0.00003*****	−3.60 (−6.49–−0.74)

Legend. Values are presented as mean ± SD and observed range (minimum–maximum); n = 4 paired donor-derived PDLSC cultures. Each time point (days 3 and 7) was analyzed separately using one-way repeated-measures ANOVA (RM-ANOVA), followed by Tukey's multiple-comparisons test comparing each PBM fluence with the corresponding non-irradiated control. The overall RM-ANOVA was significant at day 3 [$F(4,12) = 7.66$, $p = 0.0026$] and day 7 [$F(4,12) = 47.30$, $p < 0.0001$]. Effect size is reported as Cohen's d_z, calculated from paired within-donor differences (PBM minus the corresponding control), with 95% confidence intervals derived from the noncentral *t* distribution; positive values indicate stimulation and negative values inhibition. *p < 0.05; ***p < 0.001. PDLSC, periodontal ligament stem cell; PBM, photobiomodulation; SD, standard deviation.

Table S2. Effect of photobiomodulation at different fluences on PDLSC mineralization under suboptimal and complete osteogenic conditions

Fluence	Mean ± SD	Min–max	Tukey-adjusted p	Cohen's d_z (95% CI)
**Suboptimal osteogenic medium (×10⁵ positive pixels/cm²)**				
Control	2.378 ± 1.752	0.599–4.157	—	—
2 J/cm²	6.052 ± 2.474	2.780–8.451	**0.00912****	3.01 (0.54–5.48)
4 J/cm²	0.512 ± 0.451	0.077–1.114	0.2027	−1.39 (−2.79–0.09)
10 J/cm²	0.275 ± 0.128	0.122–0.417	0.1366	−1.19 (−2.49–0.19)
**Complete osteogenic medium (×10⁶ positive pixels/cm²)**				
Control	2.992 ± 0.461	2.429–3.508	—	—
2 J/cm²	4.014 ± 0.458	3.438–4.535	**0.00545****	4.56 (1.04–8.17)
4 J/cm²	2.438 ± 0.404	2.083–2.971	0.123	−1.45 (−2.89–0.06)
10 J/cm²	1.956 ± 0.520	1.297–2.555	**0.00500****	−2.00 (−3.78–−0.17)

Legend. Mineralized matrix formation was quantified by Alizarin Red S positive-pixel analysis on day 21 and is presented as mean ± SD and observed range (minimum–maximum) for n = 4 paired donor-derived PDLSC cultures. Suboptimal and complete osteogenic conditions were analyzed separately using one-way repeated-measures ANOVA (RM-ANOVA), followed by Tukey's multiple-comparisons test comparing each PBM-treated group with the corresponding non-irradiated control. The overall RM-ANOVA was significant under both suboptimal [$F(3,9) = 19.31$, $p = 0.000292$] and complete osteogenic conditions [$F(3,9) = 32.27$, $p = 0.000038$]. Effect size is reported as Cohen's d_z, calculated from paired within-donor differences (PBM minus the corresponding control), with 95% confidence intervals derived from the noncentral t distribution; positive values indicate increased and negative values decreased mineralization. **$p < 0.01$.

Table S3. Statistical analysis of osteoblastic gene expression (fold changes) in osteogenic cultures

Gene	Day	Osteo Mean ± SD	Osteo Min–Max	Osteo + 2 J/cm² Mean ± SD	Osteo + 2 J/cm² Min–Max	p value (2 J vs Osteo)	Cohen's d_z (95% CI) 2 J vs Osteo	Osteo + 10 J/cm² Mean ± SD	Osteo + 10 J/cm² Min–Max	p value (10 J vs Osteo)	Cohen's d_z (95% CI) 10 J vs Osteo
RUNX2	3	2.20 ± 0.52	1.60–2.80	2.98 ± 0.58	2.20–3.60	0.2259	0.71 (−0.45–1.79)	0.70 ± 0.45	0.10–1.20	**0.0256***	−1.85 (−3.52–−0.11)
RUNX2	7	3.48 ± 0.66	2.70–4.20	4.80 ± 0.75	3.90–5.70	**0.0448***	2.90 (0.50–5.29)	0.90 ± 0.68	0.00–1.60	**0.00208****	−2.36 (−4.38–−0.31)
RUNX2	15	1.15 ± 0.55	0.60–1.80	0.58 ± 0.59	0.00–1.30	0.5390	−0.52 (−1.54–0.57)	2.15 ± 0.77	1.40–3.00	0.2073	2.31 (0.29–4.30)
BMP2	3	5.30 ± 1.08	4.20–6.80	10.35 ± 2.00	7.60–12.40	**0.00316****	2.75 (0.45–5.04)	2.40 ± 0.63	1.80–3.20	**0.0400***	−3.22 (−5.83–−0.61)
BMP2	7	6.33 ± 0.99	5.10–7.40	14.78 ± 4.88	8.80–20.60	**0.00571****	2.14 (0.23–4.00)	1.83 ± 0.86	0.80–2.70	0.0814	−4.89 (−8.74–−1.14)
BMP2	15	2.53 ± 1.28	1.10–4.20	2.98 ± 2.00	1.40–5.80	0.922	0.15 (−0.85–1.12)	1.70 ± 0.74	0.80–2.60	0.768	−0.46 (−1.46–0.62)
SPP1	3	2.55 ± 0.85	1.60–3.60	5.23 ± 1.22	3.60–6.20	**0.0472***	1.38 (−0.10–2.77)	1.38 ± 0.98	0.70–2.80	0.4130	−1.88 (−3.58–−0.12)
SPP1	7	10.85 ± 3.48	6.40–14.60	18.80 ± 4.94	13.90–25.00	**0.0403***	1.24 (−0.16–2.55)	0.85 ± 0.62	0.00–1.40	**0.0153***	−3.17 (−5.76–−0.60)
SPP1	15	3.55 ± 1.83	1.60–5.80	1.50 ± 0.89	0.90–2.80	0.1146	−0.94 (−2.11–0.32)	1.63 ± 0.72	0.90–2.60	0.1383	−1.23 (−2.55–0.17)
TGFB1	3	2.38 ± 1.01	1.40–3.70	8.78 ± 2.01	6.30–11.00	**0.00354****	2.12 (0.22–3.98)	0.55 ± 0.72	0.00–1.60	0.3242	−2.66 (−4.89–−0.42)
TGFB1	7	3.75 ± 1.27	2.00–5.00	11.75 ± 2.54	8.80–14.20	**0.00685****	2.16 (0.24–4.05)	3.85 ± 1.86	2.00–6.20	0.9980	0.05 (−0.94–1.03)
TGFB1	15	2.38 ± 0.85	1.60–3.30	6.35 ± 1.48	5.00–8.40	**0.0171***	1.76 (0.07–3.38)	3.70 ± 1.88	2.20–6.40	0.4322	0.57 (−0.54–1.60)
COL1A1	3	0.88 ± 0.63	0.40–1.80	1.03 ± 0.31	0.60–1.30	0.9473	0.16 (−0.84–1.14)	0.90 ± 0.72	0.20–1.90	0.9985	0.02 (−0.96–1.00)
COL1A1	7	3.40 ± 0.73	2.60–4.20	5.50 ± 1.36	3.50–6.50	**0.0199***	1.56 (−0.01–3.06)	1.53 ± 0.34	1.20–2.00	**0.0321***	−3.76 (−6.77–−0.79)

Legend: Relative mRNA levels are expressed as fold change relative to the corresponding basal control, normalized to 1. Data are presented as mean ± SD and range (minimum–maximum) for n = 4 paired PDLSC lines. At each time point (days 3, 7, and 15), Osteo (PDLSCs induced for osteoblastic differentiation) and the corresponding PBM-treated cultures (2 or 10 J/cm²) were analyzed separately using one-way repeated-measures ANOVA (RM-ANOVA), followed by Tukey's multiple-comparisons test; reported p-values are Tukey-adjusted comparisons versus Osteo. Effect size is reported as Cohen's d_z for paired observations, with 95% confidence intervals of effect size calculated from the noncentral t distribution. *$p < 0.05$; **$p < 0.01$. Abbreviations: PBM, photobiomodulation; PDLSC, periodontal ligament stem cell; SD, standard deviation; *RUNX2*, runt-related transcription factor 2; *BMP2*, bone morphogenetic protein 2; *SPP1*, secreted phosphoprotein 1; *TGFB1*, transforming growth factor beta 1; *COL1A1*, collagen type I alpha 1 chain; *ALPL*, alkaline phosphatase, biomineralization associated; *BGLAP*, bone gamma-carboxyglutamate protein.

Table S4. Statistical analysis of secreted biomolecules in osteoblastic cultures

Biomolecule	Day	Osteo Mean ± SD	Osteo Min–Max	Osteo + 2 J/cm² Mean ± SD	Osteo + 2 J/cm² Min–Max	t(2)	p value	Cohen's d_z (95% CI)
**BMP-2**	3	915.75 ± 119.72	796.03–1035.47	1342.00 ± 281.86	1060.14–1623.86	4.553	**0.0450***	2.63 (0.04–5.25)
**BMP-2**	7	96.50 ± 36.48	69.45–137.99	186.50 ± 43.49	147.98–233.66	7.150	**0.0190***	4.13 (0.40–8.06)
**BMP-2**	15	62.75 ± 27.63	42.26–94.17	31.50 ± 10.25	23.90–43.16	−3.114	0.0895	−1.80 (−3.74–0.21)
**OPG**	3	21,847.00 ± 6,154.63	16,435–28,542	34,590.33 ± 3,797.93	30,826–38,421	4.764	**0.0414***	2.75 (0.07–5.48)
**OPG**	7	80,264.33 ± 21,665.62	56,879–99,654	135,117.33 ± 33,183.58	108,650–172,346	5.753	**0.0289***	3.32 (0.21–6.54)
**OPG**	15	97,557.67 ± 34,703.66	65,746–134,567	140,278.67 ± 42,969.19	106,540–188,654	1.051	0.4035	0.61 (−0.70–1.81)
**PDGF-BB**	3	256.67 ± 139.75	126–404	1363.00 ± 353.11	1084–1760	4.561	**0.0449***	2.63 (0.04–5.26)
**PDGF-BB**	7	90.67 ± 60.70	33–154	194.33 ± 54.37	150–255	14.693	**0.0046****	8.48 (1.23–16.36)
**PDGF-BB**	15	56.33 ± 28.18	34–88	117.67 ± 35.95	89–158	1.726	0.2264	1.00 (−0.51–2.39)
**ALP**	3	144.67 ± 59.14	88–206	97.67 ± 64.70	27–154	−0.659	0.5778	−0.38 (−1.52–0.84)
**ALP**	7	140.00 ± 56.32	78–188	142.33 ± 72.21	65–208	0.032	0.9774	0.02 (−1.12–1.15)
**ALP**	15	223.00 ± 38.30	190–265	300.67 ± 45.57	265–352	16.156	**0.0038****	9.33 (1.37–17.97)
**Leptin**	3	35.33 ± 16.65	22–54	60.33 ± 20.40	38–78	1.610	0.2486	0.93 (−0.54–2.28)
**Leptin**	7	124.67 ± 60.52	65–186	195.33 ± 26.84	165–216	1.478	0.2774	0.85 (−0.58–2.17)
**Leptin**	15	195.33 ± 89.67	124–296	610.00 ± 193.63	426–812	5.278	**0.0341***	3.05 (0.15–6.03)

Legend: Data are presented as mean ± SD and range (minimum–maximum) for n = 3 paired biological replicates. At each time point, Osteo (PDLSCs induced for osteoblast differentiation) (D1, D2 and D5 PDLSC cultures) and Osteo + photobiomodulation (PBM; 2 J/cm²) cultures originating from the corresponding biological replicate were paired and compared using a two-tailed paired Student's t-test. Statistical analyses were performed independently at each time point (days 3, 7, and 15) because the secretion pattern of the analyzed biomolecules is time-dependent. Effect size is reported as Cohen's d_z for paired observations, with 95% confidence intervals of Cohen's d_z calculated from the noncentral *t* distribution. *p < 0.05; **p < 0.01. Abbreviations: ALP, alkaline phosphatase; BMP-2, bone morphogenetic protein 2; OPG, osteoprotegerin; PBM, photobiomodulation; PDGF-BB, platelet-derived growth factor-BB; SD, standard deviation.
